# Evolutionary changes between pre- and post-vaccine South African group A G2P[4] rotavirus strains, 2003–2017

**DOI:** 10.1099/mgen.0.000809

**Published:** 2022-04-21

**Authors:** Peter N. Mwangi, Nicola A. Page, Mapaseka L. Seheri, M. Jeffrey Mphahlele, Sandrama Nadan, Mathew D. Esona, Benjamin Kumwenda, Arox W. Kamng’ona, Celeste M. Donato, Duncan A. Steele, Valantine N. Ndze, Francis E. Dennis, Khuzwayo C. Jere, Martin M. Nyaga

**Affiliations:** ^1^​ Next Generation Sequencing Unit and Division of Virology, Faculty of Health Sciences, University of the Free State, Bloemfontein 9300, South Africa; ^2^​ Centre for Enteric Disease, National Institute for Communicable Diseases, Private Bag X4, Sandringham, 2131, Johannesburg, South Africa; ^3^​ Department of Medical Virology, Faculty of Health Sciences, University of Pretoria, Private Bag X323, Arcadia, 0007, Pretoria, South Africa; ^4^​ Diarrheal Pathogens Research Unit, Sefako Makgatho Health Sciences University, Medunsa 0204, Pretoria, South Africa; ^5^​ Office of the Deputy Vice Chancellor for Research and Innovation, North-West University, Potchefstroom 2351, South Africa; ^6^​ South African Medical Research Council, Pretoria 0001, South Africa; ^7^​ Department of Biomedical Sciences, School of Life Sciences and Applied Health Professions, Kamuzu University of Health Sciences, Private Bag 360, Chichiri, Blantyre 3, Malawi; ^8^​ Enteric Diseases Group, Murdoch Children’s Research Institute, 50 Flemington Road, Parkville, Melboune 3052, Australia; ^9^​ Department of Paediatrics, the University of Melbourne, Parkville 3010, Australia; ^10^​ Department of Medical Laboratory Sciences, School of Life Sciences and Applied Health Professions, Kamuzu University of Health Sciences, Private Bag 360, Chichiri, Blantyre3, Malawi; ^11^​ Faculty of Health Sciences, University of Buea, P.O Box 63 Buea, Cameroon; ^12^​ Department of Electron Microscopy and Histopathology, Noguchi Memorial Institute for Medical Research, University of Ghana, P.O Box LG581, Legon, Ghana; ^13^​ Center for Global Vaccine Research, Institute of Infection, Veterinary and Ecological Sciences, University of Liverpool, L697BE, Liverpool, UK; ^14^​ Malawi-Liverpool-Wellcome Trust Clinical Research Programme, Blantyre 312225, Malawi

**Keywords:** G2P[4] group A rotavirus strains, rotavirus, sub-lineages, whole-genome analysis

## Abstract

The transient upsurge of G2P[4] group A rotavirus (RVA) after Rotarix vaccine introduction in several countries has been a matter of concern. To gain insight into the diversity and evolution of G2P[4] strains in South Africa pre- and post-RVA vaccination introduction, whole-genome sequencing was performed for RVA positive faecal specimens collected between 2003 and 2017 and samples previously sequenced were obtained from GenBank (*n*=103; 56 pre- and 47 post-vaccine). Pre-vaccine G2 sequences predominantly clustered within sub-lineage IVa-1. In contrast, post-vaccine G2 sequences clustered mainly within sub-lineage IVa-3, whereby a radical amino acid (AA) substitution, S15F, was observed between the two sub-lineages. Pre-vaccine P[4] sequences predominantly segregated within sub-lineage IVa while post-vaccine sequences clustered mostly within sub-lineage IVb, with a radical AA substitution R162G. Both S15F and R162G occurred outside recognised antigenic sites. The AA residue at position 15 is found within the signal sequence domain of Viral Protein 7 (VP7) involved in translocation of VP7 into endoplasmic reticulum during infection process. The 162 AA residue lies within the hemagglutination domain of Viral Protein 4 (VP4) engaged in interaction with sialic acid-containing structure during attachment to the target cell. Free energy change analysis on VP7 indicated accumulation of stable point mutations in both antigenic and non-antigenic regions. The segregation of South African G2P[4] strains into pre- and post-vaccination sub-lineages is likely due to erstwhile hypothesized stepwise lineage/sub-lineage evolution of G2P[4] strains rather than RVA vaccine introduction. Our findings reinforce the need for continuous whole-genome RVA surveillance and investigation of contribution of AA substitutions in understanding the dynamic G2P[4] epidemiology.

## Data Summary

The authors confirm all supporting data, code and protocols have been provided within the article or through supplementary data files.

**Impact Statement**

The abrupt increase of G2P[4] RVA strains during the post-vaccination introduction period has generated a lot of attention, especially in Rotarix vaccine introduced jurisdictions. The G2P[4] (DS-1-like) strains have a different genotype constellation to the Rotarix vaccine (Wa-like) strain suggestive of the differential protection observed against G2P[4] strains compared to Wa-like genotype constellation strains. Although G2P[4] RVA strains have been associated with dynamic epidemiology, longitudinal whole-genome based studies on this genotype have been scarce. Amino acid substitutions outside antigenic sites are generally overlooked as they are assumed not to have much evolutionary impact. Here, in our analysis of South African pre- and post-vaccine G2P[4] strains, we observed AA substitutions outside antigenic sites that appeared to define pre- and post-vaccine sub-lineages, especially on the outer capsid proteins, VP7 and VP4. Our study findings recommend an exhaustive investigation of AA substitutions outside protein antigen sites to gain insight in understanding G2P[4] epidemiology during the post-vaccination introduction era.

## Introduction

Group A rotavirus (RVA) is the primary etiological agent of acute gastroenteritis (AGE) and resulted in approximately 128 500 deaths (95 % uncertainty interval [UI], 104 500–155 600) in under-five children in 2016 [1]. Rotavirus belongs to the *Reoviridae* family and its genome comprises 11 genome segments which encode six structural proteins and five or six non-structural proteins (NSP) [2]. A binary classification scheme has traditionally been used to classify RVA into G and P types based on the properties of the outer capsid proteins, VP7 (denoted G, for its glycoprotein property) and VP4 (denoted P, for its property as a protease-sensitive protein) [3]. However, to comprehensively decipher the RVA diversity, the binary (G/P) classification system was further extended to include the other nine genome segments [4]. In the whole-genome classification scheme, a genotype is assigned to each of the 11 genome segments expressed as Gx-P[x]-Ix-Rx-Cx-Mx-Ax-Nx-Tx-Ex-Hx, whereby x is an integer and defines RVA VP7-VP4-VP6-VP1-VP2-VP3-NSP1-NSP2-NSP3-NSP4-NSP5/6, respectively [4]. The whole-genome classification also provided the basis for the three established RVA genogroups: Wa-like (genotype I constellation), DS-1-like (genotype II constellation) and AU-1-like (genotype III constellation) [4]. Nucleotide (NT) sequencing facilitated by next-generation sequencing (NGS) technologies has uncovered substantial genetic diversity among RVA, with 41 G, 57 P, 31 I, 27 R, 23 C, 23 M, 38 A, 27 N, 27 T, 31 E and 27 h genotypes reported to date [5].

The commonly detected human RVA strains globally are: G1P[8], G2P[4], G3P[8], G4P[8], G9P[8] and G12P[8] [6]. With the exception of DS-1-like G1P[8] and equine-like G3P[8] strains, the typical G1P[8], G3P[8] and G12P[8] belong to the Wa-like genogroup [7], while the G2P[4] typically possesses the DS-1-like genogroup and its predominance in certain geographical regions is well documented [8–11]. Four RVA vaccines: Rotarix (GlaxoSmithKline, Rixenstart, Belgium), Rotasiil (Serum Institute of India, India), RotaTeq (Merck and Co, USA) and Rotavac (Bharat Biotech, India) have been prequalified by World Health Organization (WHO) for global use [12]. The G1P[8]-based Rotarix vaccine offers heterotypic strain protection although higher effectiveness was described against G1P[8] strains compared with G2P[4] strains in some settings [13, 14]. Rotavirus vaccination and, in part, improvements in socio-economic factors such as safe water, sanitation and access to health care have led to a significant reduction of RVA mortality (from ~500 000 during the pre-vaccine era to ~128 000 during the post-vaccine era) globally [1, 15]. South Africa introduced the Rotarix vaccine in August 2009 [16] and RVA vaccination has reduced RVA hospitalizations by approximately 60 %, based on laboratory-confirmed results during post-vaccine introduction surveillance [17, 18].

Although RVA vaccination has reduced the burden of RVA disease, there are concerns of potential vaccine-induced selective pressure on the circulation of RVA strains [19]. The G2P[4] strains typically belong to a different genogroup with a different ancestral lineage compared to G1P[8], G3P[8], G4P[8], G9P[8] and G12P[8] strains and have been a matter of concern during the post-vaccination period [20]. In terms of diversity, G2 strains follow temporal evolutionary patterns as reported by a study that analysed VP7 sequences of G2 RVA spanning over a 35-year-period [8]. In that study, the G2 strains belonging to lineage I (G2-I), including the prototype strain, DS-1, were not detected beyond 1990 while G2 strains that clustered in lineage II (G2-II) circulated between 1991–2005. The G2 strains from 2001 to 2009 clustered mainly in lineage IV, within two distinct sub-lineages IVa-1 (which emerged in the 1990s and were detected globally) and IVa-3 (which emerged in the 2000s and spread globally) [8].

There has been a transient increase in detection of G2P[4] strains reported in several countries such as Australia, Belgium, Brazil, Japan, Saudi Arabia and Venezuela after Rotarix introduction [9, 10, 21–24]. Following Rotarix introduction in Malawi in 2012, G2 RVA (associated with either P[4], P[6] or P[8]), predominated during the early post-vaccine era and remained highly prevalent until 2018 [25]. In Zambia, a 4 year G2P[4] prevalence period in post-Rotarix introduction was reported [26]. In Kenya, G2P[4] prevalence increased from 2–17% post-Rotarix introduction [27]. The G2P[4] strains were most prevalent in 2013 in Botswana after Rotarix vaccine introduction [28]. South African G2 strains dominated alongside G1 strains in 1984, 1990 and 1993 and were also reported to exhibit a ten-year cyclic pattern with major epidemics in 1987 and 1997 [29]. Cyclic peaks of G2P[4] strains have also been reported in other studies [30–32] and the G2 lineages have been predicted to arise globally every 7 years [33]. A South African study reported three major lineages for G2 strains: one lineage involving strains collected before 1987, a second lineage comprised strains between 1988–1994 and a third lineage consisted of strains collected from 1995 [29]. Non-G1P[8] rotavirus strains increased significantly from 33 % during pre-vaccine period (2009) to 52 % during post-vaccine period (2012–2014) in South Africa [34]. The G2P[4] strains peaked and predominated in all surveillance sites in South Africa in 2013 [34].

It is unresolved whether the increased detection during the post-vaccine period of non-G1P[8] strains such as G2P[4] strains, which have different genetic backbone to the G1P[8] strain (upon which the Rotarix vaccine is based), is primarily due to the inherent RVA evolutionary mechanisms or could be vaccine-induced. The evolution of G2P[4] strains has been hypothesized to be a series of stepwise changes in lineages at whole-genome level [35]. Since there is a scarcity of whole-genome based studies of G2P[4] RVA strains in the African continent and considering that vaccine-induced selective pressure is subtle to describe within one or 2 years of vaccine introduction, whole-genome based longitudinal studies are essential to assess the impact of RVA vaccines on the evolution of RVA. Therefore, this study analysed G2P[4] strains collected between 2003–2017 in South Africa.

## Methods

### Strain description and conventional genotyping

Archival rotavirus positive stool specimens (*n*=98) were retrieved from the National Institute of Communicable Diseases (NICD), Johannesburg, South Africa (*n*=59) and from the Diarrhoeal Pathogens Research Unit (DPRU), Pretoria, South Africa (*n*=39) (Table S1, available in the online version of this article). The stool samples originated from hospital-based sentinel surveillance sites namely: Dr George Mukhari Hospital (Gauteng/North West Province), Chris Hani Baragwanath Academic Hospital (Gauteng Province), Mapulaneng Hospital (Mpumalanga Province), Matikwane Hospital (Mpumalanga Province), Edendale Hospital (KwaZulu Natal Province), Ngwelezane Hospital (KwaZulu Natal Province) and Red Cross Children’s Hospital (Western Cape Province). The stool samples were collected between 2003–2017 and were previously characterized as G2P[4] by conventional genotyping methods [36]. Briefly, viral double-stranded ribononucleic acid (dsRNA) was extracted from 10 % faecal suspension using the QIAamp viral RNA extraction method (Qiagen, Hilden, Germany). The extracted RNA was reverse transcribed and amplified using consensus primer pairs Con2/Con3 and sBeg/End9 as described previously [37, 38]. The resulting complementary deoxyribonucleic acid (cDNA) template was used for G and P typing using hemi-nested reverse transcription polymerase chain reaction (RT-PCR) amplification of the genes encoding the VP7 and VP4 [37, 38].

### Rotavirus RNA extraction and purification

Extraction of rotavirus dsRNA was performed as described previously [39]. Briefly, a faecal suspension was prepared by adding a pea size (approximately 100 mg) stool sample into 200 µl phosphate-buffered saline (PBS) solution, 0.01M, pH (Sigma-Aldrich, St Louis, MO, USA). The faecal suspension was vortexed for 10 s and let to stand at room temperature for 10 min. A volume of 300 µl supernatant was added to 900 µl TRI-Reagent-LS (Molecular Research Centre, Cincinnati, OH, USA) and then the solution was vortexed for 10 s and let to stand for 10 min at room temperature. A volume of 300 µl chloroform (Sigma-Aldrich, St Louis, MO, USA) was added to the solution containing TRI-Reagent-LS (Molecular Research Centre, Cincinnati, OH, USA) and faecal supernatant. The solution was vortexed for 10 s and let to stand for 5 min at room temperature. Then centrifugation was performed for 18 000 ***g*** for 20 min at 4 °C. After centrifugation, the aqueous supernatant was removed and added to 700 µl of ice-cold isopropanol (Sigma-Aldrich, St Louis, MO, USA). The solution was incubated at room temperature for 20 min to allow precipitation by isopropanol. Further, centrifugation was performed at 18 000 ***g*** for 30 min at room temperature. A 5 µl aliquot of dsRNA was electrophoresed in 1 % 0.5 X Tris-borate ethylenediamine tetraacetic acid (TBE) agarose (Bioline, UK) gel stained with Pronasafe (Condalab, UK) for 1 h at 95 volts. The dsRNA bands were then visualized on an ultraviolet (UV) transilluminator (Syngene, Cambridge, UK). The extracted RNA was incubated in 8M lithium chloride (Sigma-Aldrich, St Louis, MO, USA) for 16 h at 4 °C to enrich for rotavirus dsRNA [40] and then subsequently purified using the MinElute PCR purification kit (Qiagen, Hilden, Germany).

### cDNA synthesis and whole-genome sequencing

The extracted viral dsRNA was subjected to cDNA synthesis using the Maxima H Minus Double Stranded Synthesis Kit (Thermo Fisher Scientific, Waltham, MA, USA), as described previously [41]. The DNA libraries were prepared utilizing the NexteraXT DNA Library Preparation Kit (Illumina, San Diego, CA, USA) as per the manufacturer’s instructions. The Illumina MiSeq platform (Illumina, San Diego, CA, USA) at the University of the Free State - Next Generation Sequencing (UFS-NGS) Unit was utilized to perform paired-end NT sequencing (301×2) using a MiSeq Reagent Kit v3.

### Genome assembly

Quality control analysis on the raw data was performed using FASTQC [42]. Briefly, upon loading the raw sequence files in FASTQ format, FASTQC processed the data by a series of analysis modules then generated a quality control (QC) html output report showing a summary of the run modules. Alongside each analysed module, a flag of ‘Passed’, ‘Warn’ or ‘Fail’ was assigned. The ‘per base sequence quality plot’ module was assessed to consider data files with a distribution of Q30 quality score (99.9 % accuracy of the base calling at a particular position) for subsequent analysis. Illumina read ends (adapter sequences) were trimmed from the raw FASTQ sequence data using BBDuk trimmer (https://sourceforge.net/projects/bbmap/) which is embedded in Geneious Prime software, version 2020.1.1 [43]. The trimmed reads were mapped to complete nucleotide sequences of the prototype DS-1-like G2P[4] reference strain, RVA/Human-tc/USA/DS-1/1976 /G2P[4] (accession numbers HQ650116-HQ650126) using Geneious Read Mapper 6.0.3 with the Medium Sensitivity and five-times iterative fine-tuning parameters [43]. The Total Quality consensus calling was performed using the Geneious Consensus Tool by selecting 60 % highest quality threshold [43]. Regions of low coverage (<200) were annotated using the Annotate and Predict Tool in Geneious Prime version 2020.1.1. Briefly, the parameter options ‘Find regions with coverage below’ and the ‘Standard deviation from mean=2’ were selected and a coverage annotated track indicating the annotated regions was then generated to aid in the consensus calling.

### Generation of whole-genome constellations

The genotype of each genome segment was determined using web-based Virus Pathogen Database and Analysis Resource (ViPR) to generate the full-genome constellations for each RVA strain [44].

### Phylogenetic analysis

The Open Reading Frame (ORF) for each genome segment was aligned and comparatively analysed as described previously [39]. Sequence alignments were performed using the MUltiple Sequence Comparison by Log-Expectation (muscle) tool [45] in Molecular Evolutionary Genetic Analysis (mega) version 6 and further complementary analysis was performed using Multiple Alignment using Fast Fourier Transform (MAFFT) tool version 7 [46] in Geneious Prime 2020.1.1 [43]. Briefly, in muscle, a −400 Gap Opening Penalty and zero Gap Extension Penalty was selected, and the alignment process performed for eight iterations. The best evolutionary model was estimated using the Model Test in mega version 6 [47]. Briefly, maximum likelihood was selected as the statistical method for model selection analysis and gaps/missing data were treated by employing partial deletion with an 80 % site coverage cut-off with moderate branch swap filtering. Maximum-likelihood phylogenetic trees for each genome segment with a 1000 bootstrap support were constructed using mega version 6 [47]. Genetic distance matrices were calculated using the *p*-distance algorithm of mega version 6 [47]. The TREESUB phylogenetic programme [48] was used to estimate ancestral codon substitutions for VP7 and VP4 with the DS-1-like G2P[4] strain as the outgroup, using baseml as implemented in Phylogenetic Analysis by Maximum Likelihood (PAML) [49]. The DS-1 strain (G2P[4] serotype specificity) was isolated in 1976 in Washington D.C (USA) from a gastroenteritis patient [50]. The strain exhibited slow migration of genome segments 10 and 11 on the gels compared to other RVA strains and was regarded as the prototype of RVA strains exhibiting short electrophoretic patterns. Nonsynonymous substitutions were transcribed onto the generated phylogenies and visualized in FigTree version 1.4.4 (http://tree.bio.ed.ac.uk/software/figtree/).

### Protein structural and free energy change analyses

Protein modelling was performed using the SWISS-MODEL homology modelling server [51]. The VP7 protein data bank (pdb) structure, 3fmg.1, with X-ray diffraction resolution value of 3.40 Å, was selected from the SWISS-MODEL template library [52]. The stereochemical quality of the protein structures was assessed using the Structure Assessment Feature in SWISS-MODEL. The analysis and visualization of the three dimensional (3D) protein structures was performed in PyMOL [53]. The predicted VP7 protein structures covered AA residues 78–312. We did not perform protein structural and free energy change analysis on the VP4 protein structure as the available VP4 protein structure from the SWISS-MODEL template library covered 20 % of total ORF (158/775) [54]. The effect of the mutation(s) on the stability of the 3D protein structure was predicted using FoldX [55]. Briefly, the FoldX plug-in was installed in YASARA, a molecular graphics programme [56] where the the pdb file was opened. The FoldX Repair PDB Repair command was selected to optimize the free energy of the protein by rearranging the amino acid sidechains [55]. Then the Mutate Residues action was executed to calculate stability change upon mutation(s). The protein stability was estimated empirically, and the free energy change (ΔG) expressed in kcal mol^−1^. In this study, (ΔG) was determined as the free energy difference between a pre-vaccine strain (in this case the oldest South African strain(s)) and post-vaccine strain(s). A positive free energy change value was predicted to destabilize the resulting protein structure while a negative free energy change value was predicted to stabilize the mutant protein structure. Statistical significance for stabilizing/ destabilizing effect was determined at ±0.5 kcal mol^−1^ [55].

### Analysis of selection pressure

The suite of tools in the DataMonkey webserver were utilized for analyses of selective pressure on the 103 G2P[4] study strains [57]. The tools included: Fixed Effects Likelihood (FEL), Fast Unconstrained Bayesian AppRoximation (FUBAR) and Mixed Effects Model of Evolution (MEME) [58–60]. In order to minimize risk of bias by one analysis approach, a site was considered to be under positive selection if it was detected by all three methods.

## Results

### Nucleotide sequencing and whole-genotype constellations

The sequence data from the MiSeq instrument registered an overall Phred quality score of Q≥30. The average number of reads and the coverage depth for the 11 genome segments including the full-genome lengths and the respective open reading frames (ORF) are summarized in Table S2. All 103 South African G2P[4] strains analysed in this study had the DS-1-like (genotype II) constellation (I2-R2-C2-M2-A2-N2-T2-E2-H2).

### Phylogenetic and sequence analysis

Phylogenetic analysis was performed for all 11 genome segments. The VP7 and VP4 capsid proteins elicit neutralizing antibodies and have dominated focus in vaccine development [61], hence the in-depth analysis of these two genome segments in this study. The reference sequences that were utilized for lineage designations are provided in Supplementary Excelspreadsheet2.

### Phylogenetic and sequence analysis of VP7 and VP4 segments

**Fig. 1.**
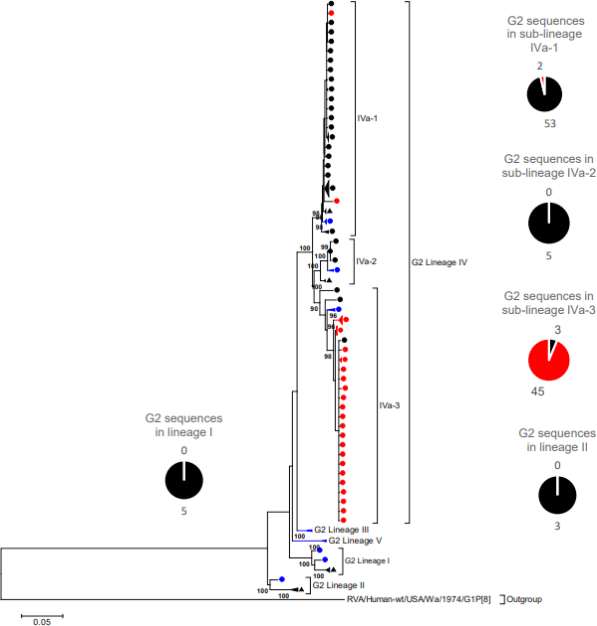
VP7 Maximum likelihood phylogenetic tree. Maximum likelihood phylogenetic tree based on the ORF of the VP7 encoding genome segment. The evolutionary model used was Tamura-3 parameter + Gamma (T92 + G). South African pre-vaccine G2 sequences are indicated with black circles while post-vaccine strains are highlighted in red circles. Thirteen previously sequenced G2 strains (from 1984 - 1996) were included for reference and are indicated with black triangles. The reference sequences used to construct the tree are indicated with blue circles. The pie-chart summarizes the number of pre- and post-vaccine sequences. Lineages are indicated in roman numerals. Only bootstrap values > 70% are shown adjacent to each branch node. The scale bar indicates the number of NT substitutions per site.

**Fig. 2.**
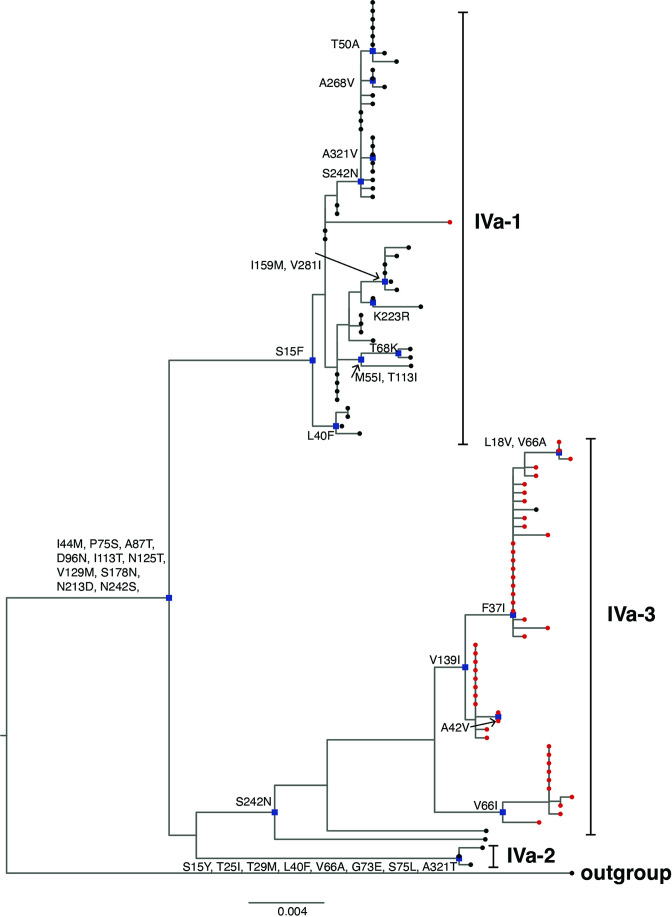
Non-synonymous amino acid substitutions in South African G2 sequences. Non-synonymous amino acid substitutions shared in each sub-lineage of G2 lineage IV where South African G2 strains in this study clustered in respect to the outgroup G2 strain, RVA/Human-tc/USA/DS-1/1976/G2P[4], are shown. South African pre-vaccine G2 sequences are indicated with black circles while post-vaccine strains are highlighted in red circles. Nodes with defining non-synonymous amino acid substitutions are highlighted in blue rectangles.

The VP7 phylogenetic tree was constructed using five previously described lineage designations [35]. South African G2 sequences (*n*=98) generated in this study clustered within lineage IV and further segregated into three sub-lineages IVa-1, IVa-2 and IVa-3 (Fig. 1). To further get insight on potential temporal evolutionary pattern of G2 sequences as reported before in literature, we included 13 South African G2 sequences retrieved from GenBank (indicated in black triangles in Fig. 1). Five of the 13 G2 sequences from 1984 to 1987 clustered within lineage I, three sequences from 1993, 1995 and 1998 clustered in lineage II, three sequences from 1996 to 1998 clustered within sub-lineage IVa-1 while two 1997 G2 sequences clustered within sub-lineage IVa-2 (Fig. 1). The G2 sequences that clustered within lineage IV shared 13 non-synonymous AA substitutions with respect to the prototype DS-1-like G2 strain found in lineage I at residues: I44M, P75S, A87T, D96N, I113T, N125T, V129M, S178N, N213D, N242S, I287V, V306I and A319T (Fig. 2). The I44M substitution lies within the cytotoxic T lymphocyte (CTL) epitope region [62] and did not result in any change in charge or polarity but the resulting residue contained a sulphur atom [63]. The six AA residues at positions 87, 96, 125, 129, 213 and 242 lie in the neutralization epitope regions 7–1 a and 7-1b, respectively [52]. The AA substitution, D96N, resulted in a change from a polar negatively charged to a polar neutral residue, while N213D substitution resulted in change from a polar neutrally charged to a polar negatively charged residue [63]. The AA residues at positions 75, 113, 178, 287, 306 and 319 lie outside the antigenic regions. Majority of pre-vaccine G2 sequences (*n*=53/69) belonged to sub-lineage IVa-1, while most of post-vaccine G2 sequences (*n*=45/47), clustered within sub-lineage IVa-3 (Fig. 1). All the G2 sequences in sub-lineage IVa-1 had a phenylalanine at position 15 while serine was observed in G2 sequences in sub-lineage IVa-3, which was a radical substitution from a non-polar to a polar AA residue [63]. Three G2 sequences that circulated in 2003 clustered within sub-lineage IVa-2 (Fig. 1) and shared AA substitutions at eight residues S15Y, T25I, T29M, L40F, V66A, G733, S75L and A321T (Fig. 2). The conservative AA substitution, L40F, lies within the CTL epitope region [62], while the rest of the AA residues lie outside the antigenic regions. The range of NT similarity between pre- and post-vaccine G2 sequences was 95.1–100 % (Table S3).

**Fig. 3.**
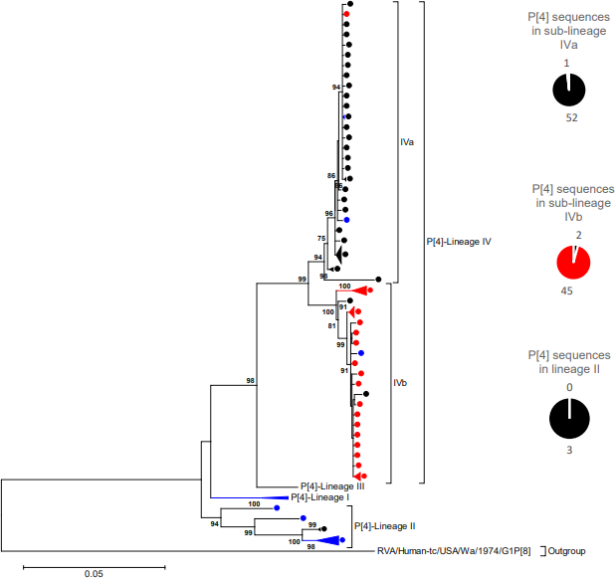
VP4 Maximum likelihood phylogenetic tree. Maximum likelihood phylogenetic tree based on the ORF of the VP4 encoding genome segment. The evolutionary model used was Tamura-3 parameter + Gamma (T92 + G). South African pre-vaccine P[4] sequences are indicated with black circles while post-vaccine sequences are highlighted in red circles. The reference sequences used to construct the tree are indicated with blue circles. The pie-chart summarizes the number of pre- and post-vaccine sequences. Lineages are indicated in roman numerals. Only bootstrap values > 70% are shown adjacent to each branch node. The scale bar indicates the number of NT substitutions per site.

**Fig. 4.**
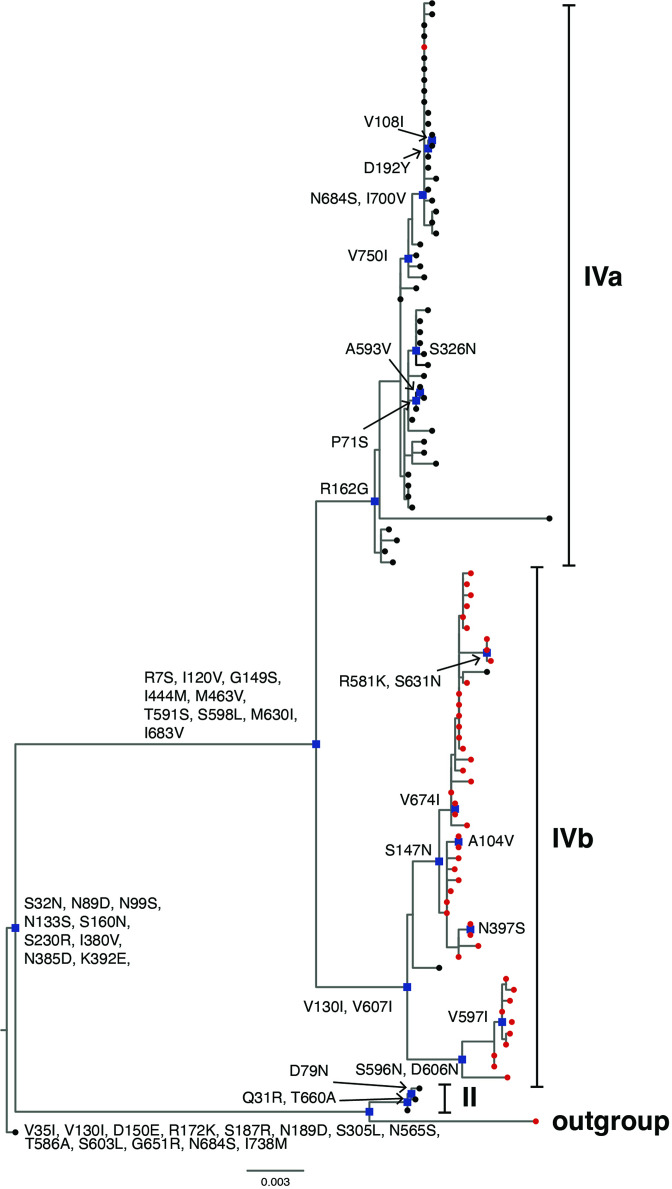
Non-synonymousamino acid substitutions shared in each sub-lineage of G2 lineage IV where South African P[4] strains in this study clustered in respect to the outgroup P[4] strain, RVA/Human-tc/USA/DS-1/1976/G2P[4], are shown. South African pre-vaccine G2 sequences are indicated with black circles while post-vaccine strains are highlighted in red circles. Nodes with defining non-synonymous amino acid substitutions are highlighted in blue rectangles.

The South African P[4] rotavirus sequences clustered within lineage II and IV of previously described lineage designations [8] (Fig. 3). Lineage II comprised three sequences that circulated in 2003 while 100 P[4] strains clustered in lineage IV (Fig. 3). All the P[4] sequences in lineage II shared non-synonymous AA substitutions in relation to the prototype DS-1-like P[4] strain at 13 residues; V35I, V130I, D150E, R172K, S187R, N189D, S305L, N565S, T586A, S603L, G651R, N684S and I178M (Fig. 4). The conservative AA substitution, D150E, lies within 8–1 a epitope region [64] while the other 12 residues reside outside antigenic regions. South African P[4] sequences in lineage IV segregated further into sub-lineages, herein defined as IVa and IVb (Fig. 2). All the P[4] sequences in lineage IV shared AA substitutions at nine residues (R7S, I120V, G149S, I444M, M463V, T591S, S598l, M630I and I683V) in relation to the prototype DS-1-like P[4] strain, which occurred in non-antigenic regions (Fig. 4). Sub-lineage IVa comprised predominantly pre-vaccine sequences that shared a glycine at position 162 while sub-lineage IVb consisted mainly of post-vaccine sequences that shared an arginine at that particular position (Fig. 4). The R162G was a radical AA substitution that occurred in the non-antigenic region and involved a change from a polar positively charged to a non-polar neutrally charged AA residue [63]. The NT similarity between pre- and post-vaccination P[4] sequences ranged from 92.0–100 % (Table S3). There were no consistently conserved AA differences in recognized antigenic regions in VP7 and VP4 between pre- vaccine and post-vaccine sequences (Supplementary Excelspreadsheet3).

### Phylogenetic and sequence analysis of VP1 - VP3, VP6 and NSP1 - NSP5 segments

The lineage attribution for the non-G and non-P genome segments was performed using a previously proposed lineage framework [65]. South African R2 sequences (*n*=100) clustered within lineage V and further segregated into pre- and post-vaccination sub-lineages while three sequences from 2003 clustered within lineage VI (Fig. S1). The C2 sequences clustered within lineage IVa (Fig. S2). The VP3 genome sequences segregated into lineage V, which comprised both pre-and post-vaccine sequences, while lineage VII predominantly consisted of post-vaccine M2 sequences (Fig. S3). The VP6 gene sequences clustered within lineage V whereby pre-vaccine sequences clustered mainly in sub-lineage Va while post-vaccine sequences dispersed into sub-lineage Vb (Fig. S4). Pre and post-vaccine sub-lineages were observed for the NSP1 gene sequences lineage IVa (Fig. S5). The N2 sequences clustered within lineage V (Fig. S6) while NSP3 genome sequences belonged to lineage V (Fig. S7). The NSP4 genome sequences were dispersed amongst four lineages: lineage V and VII, which comprised mainly pre-vaccine sequences, while lineage VI and XXIII consisted primarily of post-vaccine sequences (Fig. S8). The NSP5 phylogenetic tree did not resolve and the bootstrap was insufficient to separate the sequences into distinct sub-lineages (Fig. S9). We observed a disproportionate occurrence of AA mutations at several sites in the VP1, VP3, VP6, NSP1, NSP2 and NSP4 encoding genome segments and summarized the information including the functional roles of the domains where the disparities were identified in Tables S4–S9.

### Structural and free energy change analyses of VP7

**Fig. 5.**
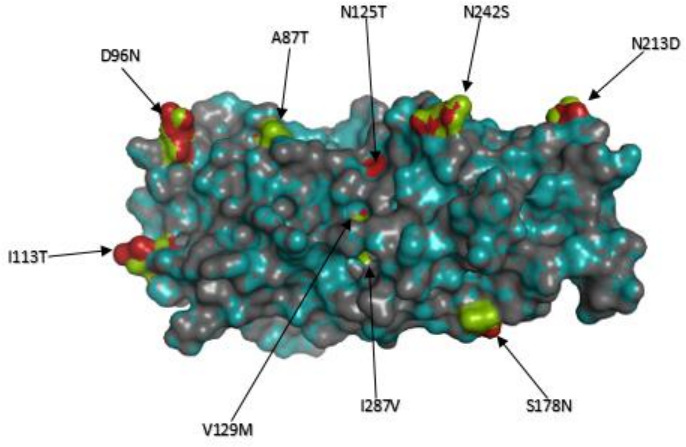
Protein structural and free energy change analysis of VP7. The superimposed VP7 protein structure models of strain, RVA/Human-wt/ZAF/UFS-NGS-NICD18920/2017/G2P[4], representative of the current post-vaccine G2P[4] strains with that of an old G2 South African strain, RVA/Human/ZAF/SA405GR/87/1987/G2P[4]. The observed AA substitutions, A87T, D96N, I113T, N125T, V129M, S178N, N213D, N242S and I287V were shared by G2 strains in lineage IV, where all the South African G2 strains in this study clustered, in relation to the G2 strains in lineage I where a 1987 G2 strain RVA/Human/ZAF/SA405GR/87/1987/G2P[4] and also the prototype DS-1-like strain, RVA/Human-tc/USA/DS-1/1976/G2P[4], clustered. The free energy change difference after mutations was -1.592 kcal/mol. The structure of the pre-vaccine strain is in deep-teal while that of the post-vaccine strain is dark gray. The AA residues are represented in green and firebrick red to examine the structural conformation of the protein structure. The AA residues highlighted in firebrick red represents the pre-vaccine strain while that in green represents the post-vaccine strain.

To get an insight on the potential structural conformation difference between post-vaccine G2 sequences and five old G2 sequences that circulated in South Africa between 1984 - 1987, we superimposed VP7 protein structures from the two time periods (Fig. 5, Table 1). The root-mean-square deviation (RMSD) value ranged from 0.017 to 0.019 indicating some level of alteration in structural conformation potentially due to the replacing amino acids (An RMSD value of 0.000 indicates absolute similarity) [66]. The predicted energy change differences after mutating the three old G2 protein structures at residues A87T, D96N, I113T, N125T, V129M, S178N, N213D, N242S and I287V D96N, V129M, S178N and N213D which defined G2 strains in lineage I and IV, ranged from −0.523 to −1.592 kcal mol^−1^, indicating stabilizing mutations (Fig. 5, Table 1).

**Table 1.** Protein structural and free energy change analysis of VP7

Pre-vaccine strain	Post-vaccine strain	Energy change in kcal/mol	Alignment value in RMSD (Å)
RVA/Human-wt/ZAF/SA296GR/84/1984 /G2P[4]	RVA/Human-wt/ZAF/UFS-NGS-NICD18920/2017 /G2P[4]	−0.523	0.018
RVA/Human-wt/ZAF/SA410GR/85/1985 /G2P[4]	RVA/Human-wt/ZAF/UFS-NGS-MRC-DPRU68/2013 /G2P[4]	−1.017	0.018
RVA/Human-wt/ZAF/SA659GR/86/1986 /G2P[4]	RVA/Human-wt/ZAF/UFS-NGS-NICD13522/2014 /G2P[4]	−0.786	0.017
RVA/Human-wt/ZAF/SA405GR/87/1987 /G2P[4]	RVA/Human-wt/ZAF/UFS-NGS-NICD15034/2015 /G2P[4]	−1.592	0.017
RVA/Human-wt/ZAF/SA514GR/87/1987 /G2P[4]	RVA/Human-wt/ZAF/UFS-NGS-NICD12041/2013 /G2P[4]	−0.528	0.019

The table includes five G2 strains that circulated in South Africa from 1984−1987 and clustered in lineage I alongside the prototype DS-1-like strain. Five post-vaccine G2 strains were randomly selected for the protein structural and free energy change analyses. Protein stability was predicted by estimating the energy change, expressed in kcal mol^−1^, between a pre-vaccine strain and post-vaccine strain. The energy change o ±0.5 was regarded significant for stabilizing or destabilizing effect. A positive (+) value indicates destabilizing effect while a negative (−) value indicates stabilizing effect. The impact of mutation on the structural conformation of the protein was assessed by superposing a pre-vaccine strain with a post-vaccine strain and the RMSD (Root Mean Square Deviation) alignment value expressed in Angstroms (Å). An RMSD value of zero indicates absolute structural alignment

### Selection pressure analysis

Selection pressure analysis by FEL, FUBAR) and MEME showed that the following AA sites were under positive selection: 14 (VP2), 7 (VP3), 7 (VP4), 13 (VP6), 254 (NSP2), 6 (NSP3) and 5 (NSP4). These positively selected sites occurred in the signal peptide regions of the genome segments apart from site 254 which occurred in an RNA-binding domain [66–71]. The rest of the codon sites in the 11 gene segments were undergoing purifying selection (Table 2).

**Table 2.** Positively selected codon sites in 103 South African G2P[4] pre- and post-RVA amino acid residues as identified by FEL, FUBAR and MEME analysis

Method	Amino acid sites in the gene segments under positive selection										
	VP1	VP2	VP3	VP4	VP6	VP7	NSP1	NSP2	NSP3	NSP4	NSP5
**MEME**	2,62,90, 97 128, 157 158, 187 332, 364 371, 406 546, 654,1033,1041	5,13, **14**, 723 869, 873 874, 877 878	**7**,25,133,153,322, 346 473, 704 788, 828 835	**7** 192, 625 627	**13**	315	10 212, 271 313	249 **254**	3, **6**, 16,27	2,3, **5**,9,10, 16,17,51,79,170	3,7
**FUBAR**	60 364	**14**, 878	**7**	**7**	**13**	66	10,11, 103 380 402	75 **254**	**6**	**5**	–
**FEL**	–	**14**	**7** 828	**7**	**13**	–	319	**254**	**6**	**5**,9	–

Bold and underlined sites are those identified by all the three methods: Fixed Effects Likelihood (FEL), Fast Unconstrained Bayesian AppRoximation (FUBAR) and Mixed Effects Model of Evolution (MEME). For MEME and FEL, statistical significance was assessed at *P*≤0.1 while for FUBAR it was assessed at posterior probability ≥0.9. The dash (-) sign indicates no site was identified. Further analysis reports are provided in the Supplementary Excelspreadsheet1.

## Discussion

The present study reports whole-genome analysis of G2P[4] RVA strains collected from South Africa between 2003–2017. The DS-1-like constellation findings exhibited by the study strains are consistent with whole-genome studies of G2P[4] strains in several countries [33, 35, 72–74]. The sub-lineage shifts that were observed post-vaccine introduction are comparable to the findings of unique sub-clusters that were identified in a detailed whole-genome analysis in Belgium, albeit in G1P[8] strains [24].

South African G2 sequences in this study shared an asparagine at antigenic position 96 (N96), a hallmark of lineage IV linked with re-emergence of G2 strains from 2000 [8]. The G2 sub-lineage IVa-3 predominantly comprised post-vaccine strains characterized by an asparagine residue at antigenic position 242 (N242) observed in most modern G2 sequences globally [8, 74]. The radical AA substitution, S15F, between G2 sequences in sub-lineage IVa-1 and IVa-3 occurred in the signal sequence domain of VP7 and mutations in this region potentially enhance translocation of VP7 into endoplasmic reticulum during infection process [75].

The majority of South African P[4] strains clustered within lineage IV which emerged during 2000s [76] and circulated worldwide as the strains gained epidemiological fitness [77]. The R162G radical AA substitution between P[4] strains in sub-lineage IVa and sub-lineage IVb lies within the hemagglutination domain of VP4 and mutation(s) in this region likely impact interaction with sialic-acid containing structure during attachment to the target cell, possibly improving the interaction [78]. We did not observe conserved AA differences between pre-vaccine and post-vaccine strains in the antigenic regions of VP7 and VP4. Therefore, it appears Rotarix introduction did not prompt significant mutations in the VP7 and VP4 antigenic regions.

Notably, the disproportionate occurrence of AA residues at particular sites in the gene segments between pre- and post-vaccine G2P[4] strains occurred in regions that potentially enhance viral infectivity of the virus. For example, in VP6, AA substitutions were observed within a region (site V281I) thought to interact with heat shock cognate protein (hsc70), a co-receptor for rotavirus entry into susceptible cells [79]. In VP3, up to nine mutations were found in the 2’5-phosphodiesterase domain which is involved in suppression of antiviral innate immunity by preventing activation of the cellular oligoadenylate synthetase (OAS)/RNase L. pathway [80]. In NSP4, a V135M mutation occurred within the enterotoxin domain that is implicated in diarrhea-induction through integrin binding and signalling, while R137Q, T139I and N140C appeared in the integrin binding domain [81].

Protein structural and free energy change analysis between old (1984–1987) pre-vaccine South African G2 strains that clustered in lineage I and post-vaccine strains in lineage IV indicated the introduction of stable point mutations in the antigenic and non-antigenic regions that potentially enhance viral fitness. The consensus selection force in the codon sites of the 11 gene segments was negative selection. This purifying selection may be a strategy to purge any resultant deleterious polymorphisms that arise due to the high random mutation rates of the error-prone RNA-dependent RNA polymerase [2]. Positive selection in the signal peptide region of the gene segments is suggested to optimize translation [82] possibly as a strategy to enhance viral fitness.

We acknowledge some limitations in this study. There was a lack of G2P[4] samples in the years 2004, 2005 and 2011 although G2P[4] RVA strains are generally reported to exhibit fluctuation patterns [33]. The unavailability of completely resolved protein structures covering the whole ORF hindered comprehensive free energy change analysis on the impact of mutation on the protein structures.

## Conclusion

The findings from our full-genome analysis of rotavirus G2P[4] strains collected from South Africa over a period of 13 years indicated AA substitutions in the non-antigenic regions in post-vaccine strains relative to pre-vaccine strains. These substitutions lead to pre- and post-vaccine RVA strains clustering in distinct sub-lineages. While it is possible to infer that AA substitutions could be a strategy to cope with population immunity generated after RVA vaccine introduction, selected AA residues were observed in the genome segments prior to vaccine introduction, suggesting that natural evolution dynamics of wild-type RVA are likely to be involved. Therefore, continuous whole-genome surveillance is required to refine our understanding of non-antigenic regions in RVA evolutionary dynamics. This will be essential to fully assess the impact of RVA vaccine introduction on the RVA strains circulating during the post-vaccine period.

## Supplementary Data

Supplementary material 1

Supplementary material 2

Supplementary material 3

Supplementary material 4

## References

[1] Troeger C, Khalil IA, Rao PC, Cao S, Blacker BF (2018). Rotavirus vaccination and the global burden of rotavirus diarrhea among children younger than 5 years. JAMA Pediatr.

[2] Estes MK, Greenberg HB, Knipe DM, Howley PM (2013). Fields Virology.

[3] Estes MK, Kapikian AZ, Knipe D, Griffin D, Lamb R, Martin M, Roizman B (2007). Fields Virology.

[4] Matthijnssens J, Ciarlet M, McDonald SM, Attoui H, Bányai K (2011). Uniformity of rotavirus strain nomenclature proposed by the Rotavirus Classification Working Group (RCWG). Arch Virol.

[5] Virus Classification (2021). Rega.kuleuven.be. https://rega.kuleuven.be/cev/viralmetagenomics/virus-classification.

[6] Dóró R, László B, Martella V, Leshem E, Gentsch J (2014). Review of global rotavirus strain prevalence data from six years post vaccine licensure surveillance: is there evidence of strain selection from vaccine pressure. Infect Genet Evol.

[7] Matthijnssens J, Van Ranst M (2012). Genotype constellation and evolution of group A rotaviruses infecting humans. Curr Opin Virol.

[8] Doan YH, Nakagomi T, Cunliffe NA, Pandey BD, Sherchand JB (2011). The occurrence of amino acid substitutions D96N and S242N in VP7 of emergent G2P [4] rotaviruses in Nepal in 2004-2005: a global and evolutionary perspective. Arch Virol.

[9] Vizzi E, Piñeros OA, Oropeza MD, Naranjo L, Suárez JA (2017). Human rotavirus strains circulating in Venezuela after vaccine introduction: predominance of G29 [P4] and reemergence of G1P [8]. Virol J.

[10] Khandoker N, Thongprachum A, Takanashi S, Okitsu S, Nishimura S (2018). Molecular epidemiology of rotavirus gastroenteritis in Japan during 2014-2015: Characterization of re-emerging G2P[4] after rotavirus vaccine introduction. J Med Virol.

[11] Thanh HD, Tran VT, Lim I, Kim W (2018). Emergence of Human G2P[4] Rotaviruses in the Post-vaccination Era in South Korea: Footprints of Multiple Interspecies Re-assortment Events. Sci Rep.

[12] WHO (2021). WHO prequalifies new rotavirus vaccine. https://www.who.int/teams/immunization-vaccines-and-biologicals/diseases/rotavirus.

[13] Braeckman T, Van Herck K, Meyer N, Pirçon J-Y, Soriano-Gabarró M (2012). Effectiveness of rotavirus vaccination in prevention of hospital admissions for rotavirus gastroenteritis among young children in Belgium: case-control study. BMJ.

[14] Bar-Zeev N, Jere KC, Bennett A, Pollock L, Tate JE (2016). Population impact and effectiveness of monovalent rotavirus vaccination in urban Malawian children 3 years after vaccine introduction: ecological and case-control analyses. Clin Infect Dis.

[15] Parashar UD, Gibson CJ, Bresee JS, Glass RI (2006). Rotavirus and severe childhood diarrhea. Emerg Infect Dis.

[16] World Health Organization (2009). Meeting of the immunization Strategic Advisory Group of Experts, April 2009—conclusions and recommendations. WER.

[17] Msimang VMY, Page N, Groome MJ, Moyes J, Cortese MM (2013). Impact of rotavirus vaccine on childhood diarrheal hospitalization after introduction into the South African public immunization program. Pediatr Infect Dis J.

[18] Steele AD, Groome MJ (2020). Measuring Rotavirus Vaccine Impact in Sub-Saharan Africa. Clin Infect Dis.

[19] Leshem E, Lopman B, Glass R, Gentsch J, Bányai K (2014). Distribution of rotavirus strains and strain-specific effectiveness of the rotavirus vaccine after its introduction: a systematic review and meta-analysis. Lancet Infect Dis.

[20] Bibera GL, Chen J, Pereira P, Benninghoff B (2020). Dynamics of G2P[4] strain evolution and rotavirus vaccination: A review of evidence for Rotarix. Vaccine.

[21] Gurgel RQ, Cuevas LE, Vieira SCF, Barros VCF, Fontes PB (2007). Predominance of rotavirus P[4]G2 in a vaccinated population, Brazil. Emerg Infect Dis.

[22] Donato C (2012). Molecular epidemiology of rotavirus in the era of vaccination. Microbiol Aust.

[23] Al-Ayed MSZ, Asaad AM, Qureshi MA, Hawan AA (2017). Epidemiology of group A rotavirus infection after the introduction of monovalent vaccine in the National Immunization Program of Saudi Arabia. J Med Virol.

[24] Zeller M, Heylen E, Tamim S, McAllen JK, Kirkness EF (2017). Comparative analysis of the Rotarix vaccine strain and G1P[8] rotaviruses detected before and after vaccine introduction in Belgium. PeerJ.

[25] Mhango C, Mandolo JJ, Chinyama E, Wachepa R, Kanjerwa O (2020). Rotavirus Genotypes in Hospitalized Children with Acute Gastroenteritis Before and After Rotavirus Vaccine Introduction in. J Infect Dis.

[26] Simwaka JC, Mpabalwani EM, Seheri M, Peenze I, Monze M (2018). Diversity of rotavirus strains circulating in children under five years of age who presented with acute gastroenteritis before and after rotavirus vaccine introduction. Vaccine.

[27] Wandera EA, Mohammad S, Bundi M, Komoto S, Nyangao J (2017). Impact of rotavirus vaccination on rotavirus and all-cause gastroenteritis in peri-urban Kenyan children. Vaccine.

[28] Mokomane M, Esona MD, Bowen MD, Tate JE, Steenhoff AP (2019). Diversity of Rotavirus Strains Circulating in Botswana before and after introduction of the Monovalent Rotavirus Vaccine. Vaccine.

[29] Page NA, Steele AD (2004). Antigenic and genetic characterization of serotype G2 human rotavirus strains from South Africa from 1984 to 1998. J Med Virol.

[30] Linhares AC, Justino MCA (2014). Rotavirus vaccination in Brazil: effectiveness and health impact seven years post-introduction. Expert Rev Vaccines.

[31] Patton JT (2012). Rotavirus diversity and evolution in the post-vaccine world. Discov Med.

[32] Pitzer VE, Patel MM, Lopman BA, Viboud C, Parashar UD (2011). Modeling rotavirus strain dynamics in developed countries to understand the potential impact of vaccination on genotype distributions. Proc Natl Acad Sci U S A.

[33] Dennis AF, McDonald SM, Payne DC, Mijatovic-Rustempasic S, Esona MD (2014). Molecular epidemiology of contemporary G2P[4] human rotaviruses cocirculating in a single U.S. community: footprints of a globally transitioning genotype. J Virol.

[34] Page NA, Seheri LM, Groome MJ, Moyes J, Walaza S (2018). Temporal association of rotavirus vaccination and genotype circulation in South Africa: Observations from 2002 to 2014. Vaccine.

[35] Doan YH, Nakagomi T, Agbemabiese CA, Nakagomi O (2015). Changes in the distribution of lineage constellations of G2P[4] Rotavirus A strains detected in Japan over 32 years (1980-2011). Infect Genet Evol.

[36] Seheri M, Nemarude L, Peenze I, Netshifhefhe L, Nyaga MM (2014). Update of rotavirus strains circulating in Africa from 2007 through 2011. Pediatr Infect Dis J.

[37] Gentsch JR, Glass RI, Woods P, Gouvea V, Gorziglia M (1992). Identification of group A rotavirus gene 4 types by polymerase chain reaction. J Clin Microbiol.

[38] Gouvea V, Glass RI, Woods P, Taniguchi K, Clark HF (1990). Polymerase chain reaction amplification and typing of rotavirus nucleic acid from stool specimens. J Clin Microbiol.

[39] Maringa WM, Mwangi PN, Simwaka J, Mpabalwani EM, Mwenda JM (2020). Molecular characterisation of a rare reassortant porcine-like G5P[6] rotavirus strain detected in an unvaccinated child in Kasama, Zambia. Pathogens.

[40] Potgieter AC, Page NA, Liebenberg J, Wright IM, Landt O (2009). Improved strategies for sequence-independent amplification and sequencing of viral double-stranded RNA genomes. J Gen Virol.

[41] Mwangi PN, Mogotsi MT, Seheri ML, Mphahlele MJ, Peenze I (2020). Whole Genome *In-Silico* Analysis of South African G1P[8] Rotavirus Strains Before and After Vaccine Introduction Over A Period of 14 Years. Vaccines (Basel).

[42] Andrews SF (2010). A quality control tool for high throughput sequence data.

[43] Kearse M, Moir R, Wilson A, Stones-Havas S, Cheung M (2012). Geneious Basic: an integrated and extendable desktop software platform for the organization and analysis of sequence data. Bioinformatics.

[44] Pickett BE, Sadat EL, Zhang Y, Noronha JM, Squires RB (2012). ViPR: an open bioinformatics database and analysis resource for virology research. Nucleic Acids Res.

[45] Edgar RC (2004). MUSCLE: multiple sequence alignment with high accuracy and high throughput. Nucleic Acids Res.

[46] Katoh K, Standley DM (2013). MAFFT multiple sequence alignment software version 7: improvements in performance and usability. Mol Biol Evol.

[47] Tamura K, Stecher G, Peterson D, Filipski A, Kumar S (2013). MEGA6: molecular evolutionary genetics analysis version 6.0. Mol Biol Evol.

[48] GitHub (2021). Tamuri/treesub [Internet]. https://github.com/tamuri/treesub.

[49] Yang Z (2007). PAML 4: phylogenetic analysis by maximum likelihood. Mol Biol Evol.

[50] Kalica AR, Greenberg HB, Espejo RT, Flores J, Wyatt RG (1981). Distinctive ribonucleic acid patterns of human rotavirus subgroups 1 and 2. Infect Immun.

[51] Waterhouse A, Bertoni M, Bienert S, Studer G, Tauriello G (2018). SWISS-MODEL: homology modelling of protein structures and complexes. Nucleic Acids Res.

[52] Aoki ST, Settembre EC, Trask SD, Greenberg HB, Harrison SC (2009). Structure of rotavirus outer-layer protein VP7 bound with a neutralizing Fab. Science.

[53] DeLano WL (2002). Pymol: An open-source molecular graphics tool. CCP4 Newsletter on protein crystallography.

[54] Blanchard H, Yu X, Coulson BS, von Itzstein M (2007). Insight into host cell carbohydrate-recognition by human and porcine rotavirus from crystal structures of the virion spike associated carbohydrate-binding domain (VP8. J Mol Biol.

[55] Van Durme J, Delgado J, Stricher F, Serrano L, Schymkowitz J (2011). A graphical interface for the FoldX forcefield. Bioinformatics.

[56] Krieger E, Vriend G (2014). YASARA View—molecular graphics for all devices—from smartphones to workstations. Bioinformatics.

[57] Weaver S, Shank SD, Spielman SJ, Li M, Muse SV (2018). Datamonkey 2.0: a modern web application for characterizing selective and other evolutionary processes. Mol Biol Evol.

[58] Kosakovsky Pond SL, Frost SDW (2005). Not so different after all: a comparison of methods for detecting amino acid sites under selection. Mol Biol Evol.

[59] Murrell B, Wertheim JO, Moola S, Weighill T, Scheffler K (2012). Detecting individual sites subject to episodic diversifying selection. PLoS Genet.

[60] Murrell B, Moola S, Mabona A, Weighill T, Sheward D (2013). FUBAR: a fast, unconstrained bayesian approximation for inferring selection. Mol Biol Evol.

[61] Burke RM, Tate JE, Kirkwood CD, Steele AD, Parashar UD (2019). Current and new rotavirus vaccines. Curr Opin Infect Dis.

[62] Franco MA, Tin C, Greenberg HB (1997). CD8+ T cells can mediate almost complete short-term and partial long-term immunity to rotavirus in mice. J Virol.

[63] Betts MJ, Russell RB (2003). Amino acid properties and consequences of substitutions. Bioinformatics for geneticists.

[64] Dormitzer PR, Sun Z-YJ, Wagner G, Harrison SC (2002). The rhesus rotavirus VP4 sialic acid binding domain has a galectin fold with a novel carbohydrate binding site. EMBO J.

[65] Agbemabiese CA, Nakagomi T, Damanka SA, Dennis FE, Lartey BL (2019). Sub-genotype phylogeny of the non-G, non-P genes of genotype 2 Rotavirus A strains. PLoS One.

[66] Maiorov VN, Crippen GM (1994). Significance of root-mean-square deviation in comparing three-dimensional structures of globular proteins. J Mol Biol.

[67] Piron M, Delaunay T, Grosclaude J, Poncet D (1999). Identification of the RNA-binding, dimerization, and eIF4GI-binding domains of rotavirus nonstructural protein NSP3. J Virol.

[68] Charpilienne A, Lepault J, Rey F, Cohen J (2002). Identification of rotavirus VP6 residues located at the interface with VP2 that are essential for capsid assembly and transcriptase activity. J Virol.

[69] Dormitzer PR, Sun Z-YJ, Wagner G, Harrison SC (2002). The rhesus rotavirus VP4 sialic acid binding domain has a galectin fold with a novel carbohydrate binding site. EMBO J.

[70] McDonald SM, Patton JT (2011). Rotavirus VP2 core shell regions critical for viral polymerase activation. J Virol.

[71] Viskovska M, Anish R, Hu L, Chow D-C, Hurwitz AM (2014). Probing the sites of interactions of rotaviral proteins involved in replication. J Virol.

[72] Donato CM, Zhang ZA, Donker NC, Kirkwood CD (2014). Characterization of G2P[4] rotavirus strains associated with increased detection in Australian states using the RotaTeq® vaccine during the 2010-2011 surveillance period. Infect Genet Evol.

[73] Nyaga MM, Stucker KM, Esona MD, Jere KC, Mwinyi B (2014). Whole-genome analyses of DS-1-like human G2P[4] and G8P[4] rotavirus strains from Eastern, Western and Southern Africa. Virus Genes.

[74] Agbemabiese CA, Nakagomi T, Doan YH, Do LP, Damanka S (2016). Genomic constellation and evolution of Ghanaian G2P[4] rotavirus strains from a global perspective. Infect Genet Evol.

[75] Stirzaker SC, Poncet D, Both GW (1990). Sequences in rotavirus glycoprotein VP7 that mediate delayed translocation and retention of the protein in the endoplasmic reticulum. J Cell Biol.

[76] Gómez MM, de Mendonça MCL, Volotão E de M, Tort LFL, da Silva MFM (2011). Rotavirus A genotype P[4]G2: genetic diversity and reassortment events among strains circulating in Brazil between 2005 and 2009. J Med Virol.

[77] Giammanco GM, Bonura F, Zeller M, Heylen E, Van Ranst M (2014). Evolution of DS-1-like human G2P[4] rotaviruses assessed by complete genome analyses. J Gen Virol.

[78] Fuentes-Pananá EM, López S, Gorziglia M, Arias CF (1995). Mapping the hemagglutination domain of rotaviruses. J Virol.

[79] Gualtero DF, Guzmán F, Acosta O, Guerrero CA (2007). Amino acid domains 280–297 of VP6 and 531–554 of VP4 are implicated in heat shock cognate protein hsc70-mediated rotavirus infection. Arch Virol.

[80] Ogden KM, Snyder MJ, Dennis AF, Patton JT (2014). Predicted structure and domain organization of rotavirus capping enzyme and innate immune antagonist VP3. J Virol.

[81] Seo NS, Zeng CQ, Hyser JM, Utama B, Crawford SE (2008). Integrins α1β1 and α2β1 are receptors for the rotavirus enterotoxin. PNAS.

[82] Akashi H (2001). Gene expression and molecular evolution. Curr Opin Genet Dev.

