# Supplementary material for: Evolutionary changes between pre- and post-vaccine South African group A G2P[4] rotavirus strains, 2003–2017
Source: Microb Genom. 2022 Apr 21;8(4):000809. doi: 10.1099/mgen.0.000809 (PMC9453071; doi:10.1099/mgen.0.000809)
Supplement: Supplementary material 1 [file mgen-8-0809-s001.pdf]

Figures S1-S9

VP1

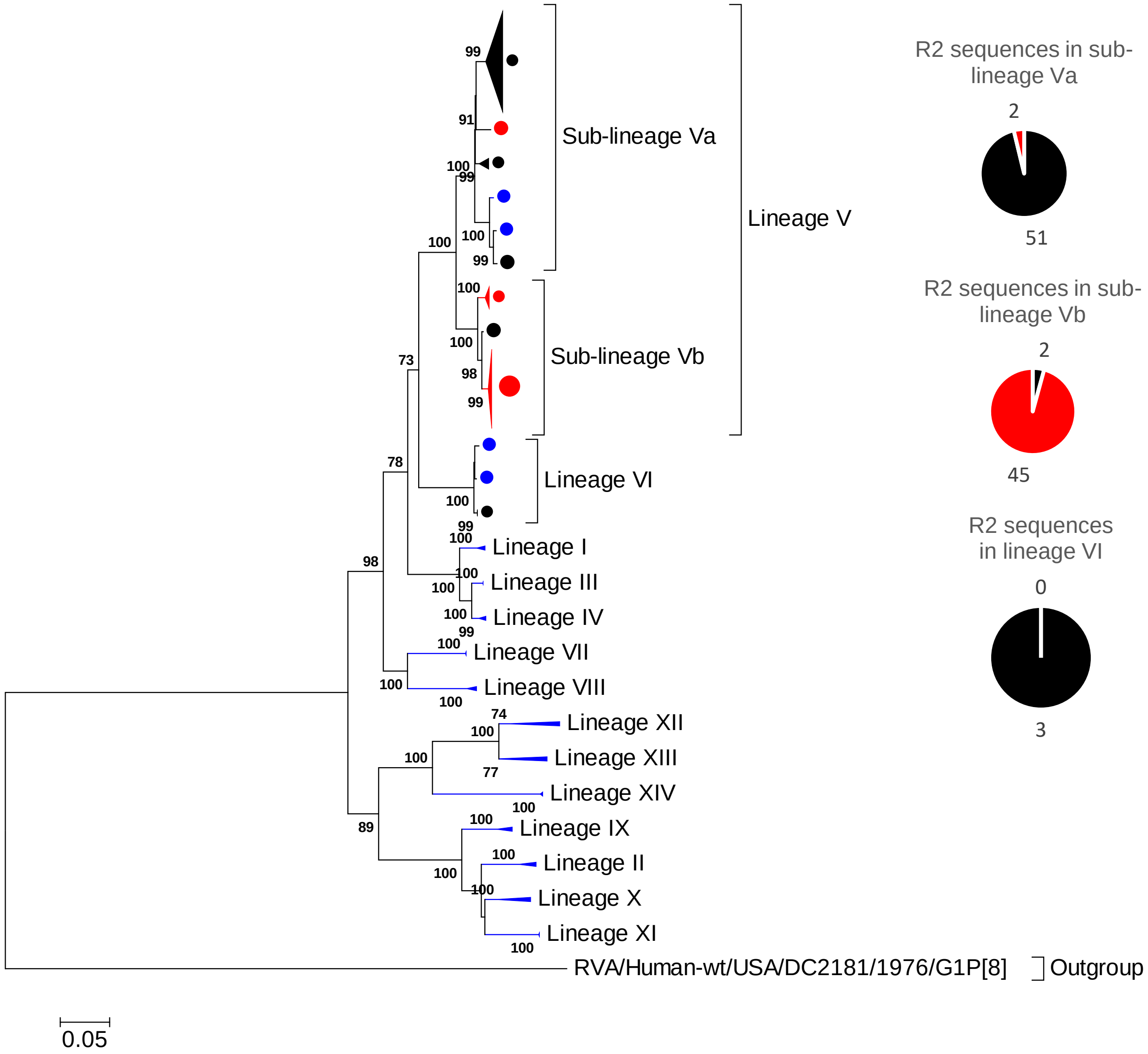

Figure S1: Maximum likelihood phylogenetic tree based on the ORF of the genome segment 1 encoding VP1. The HKY + G evolutionary model was used for phylogenetic inference. South African pre-vaccine R2 sequences are indicated with black circles while post-vaccine R2 sequences are highlighted in red circles. The reference sequences that were used to construct the tree are indicated with blue circle. The pie-chart summarizes the number of pre- and post-vaccine sequences. Lineages are indicated in roman numerals. Only bootstrap values ≥ 70% are shown adjacent to each branch node. Scale bar indicates the number of nucleotide substitutions per site.

VP2

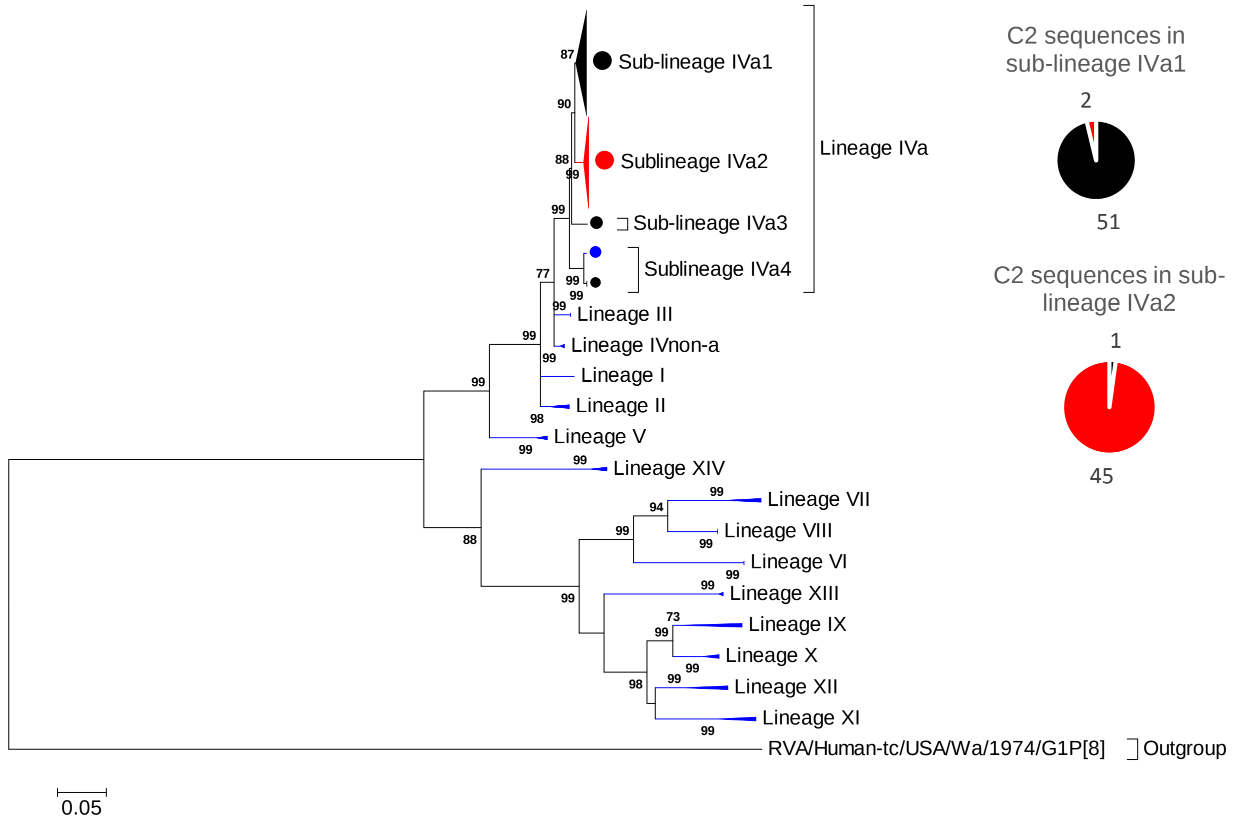

Figure S2: Maximum likelihood phylogenetic tree based on the ORF of the genome segment 2 encoding VP2. The GTR + G + I evolutionary model was used for phylogenetic inference. South African pre-vaccine C2 sequences are indicated with black circles while post-vaccine sequences are highlighted with blue circle. The pie-chart summarizes the number of pre- and post-vaccine sequences. Lineages are indicated in roman numerals. Only bootstrap values ≥ 70% are shown adjacent to each branch node. Scale bar indicates the number of nucleotide substitutions per site.

VP3

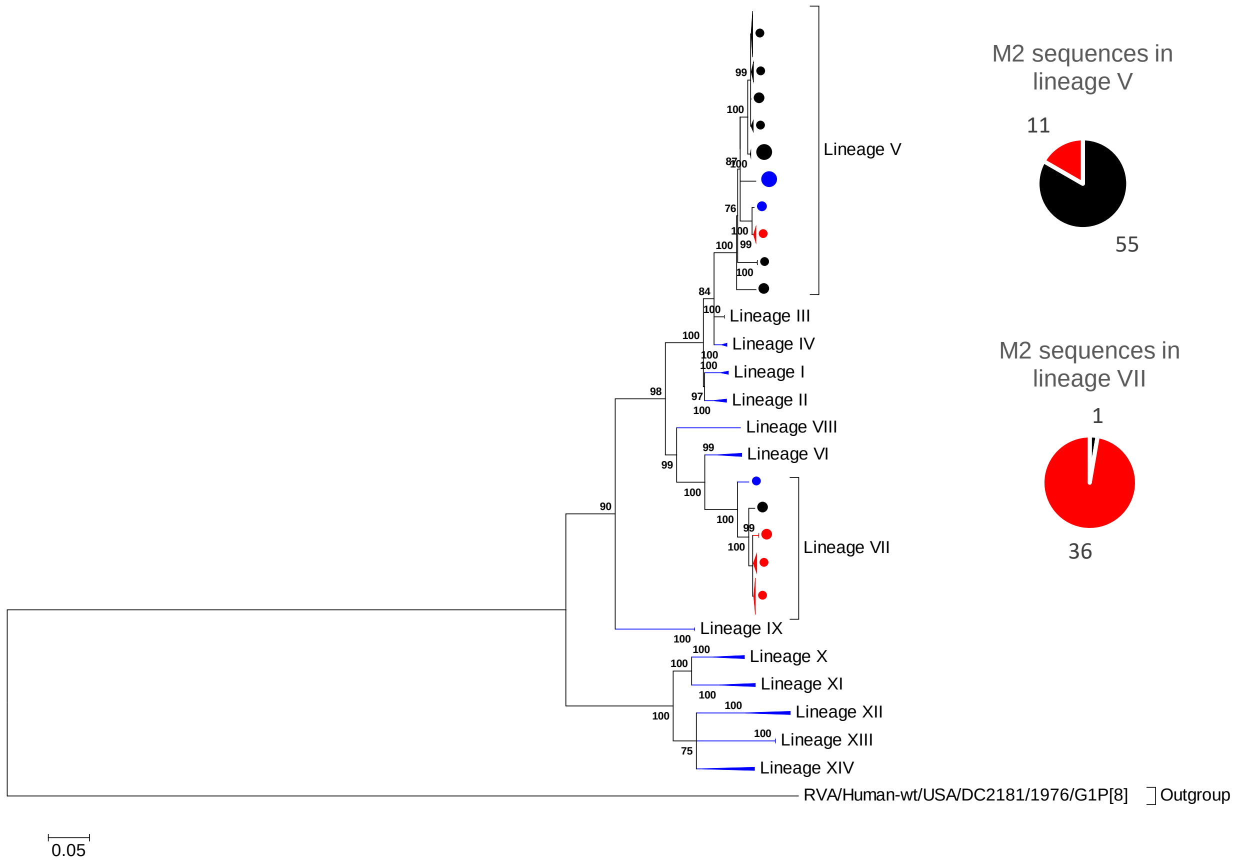

Figure S3: Maximum likelihood phylogenetic tree based on the ORF of the genome segment 3 encoding VP3. The GTR+G+I evolutionary model was used for phylogenetic inference. South African pre-vaccine M2 sequences are indicated with black circles while post-vaccine sequences are highlighted in red circles. The reference sequences that were used to construct the tree are indicated with blue circle. The pie-chart summarizes the number of pre- and post-vaccine sequences. Lineages are indicated in roman numerals. Only bootstrap values ≥ 70% are shown adjacent to each branch node. Scale bar indicates the number of nucleotide substitutions per site.

VP6

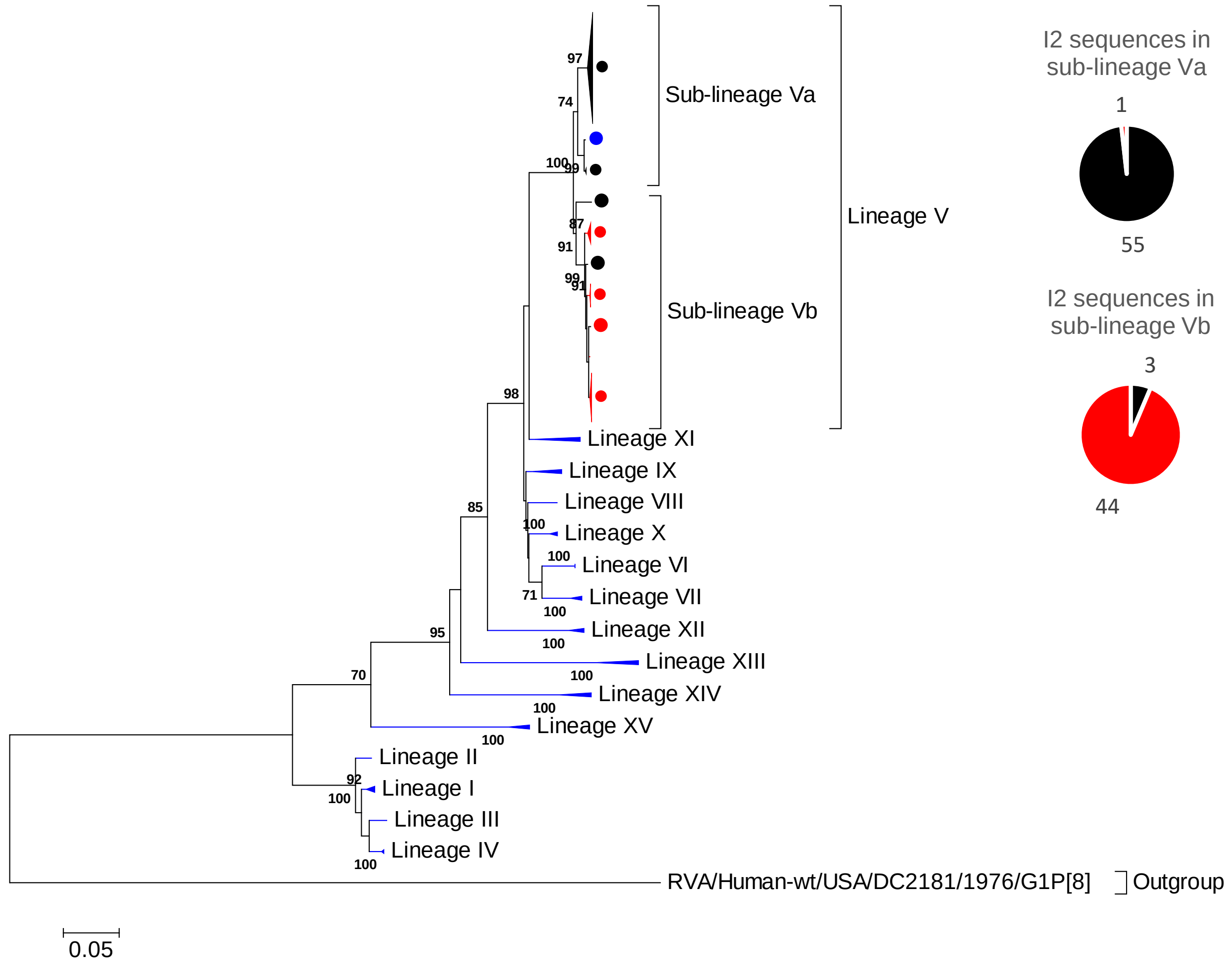

Figure S4: Maximum likelihood phylogenetic tree based on the ORF of the genome segment 6 encoding VP6. The T92 + G + I evolutionary model was used for phylogenetic inference. South African pre-vaccine I2 sequences are indicated with black circles while post-vaccine sequences are highlighted in red circles. The reference sequences that were used to construct the tree are indicated with blue circle. The pie-chart summarizes the number of pre- and post-vaccine sequences. Lineages are indicated in roman numerals. Only bootstrap values ≥ 70% are shown adjacent to each branch node. Scale bar indicates the number of nucleotide substitutions per site.

NSP1

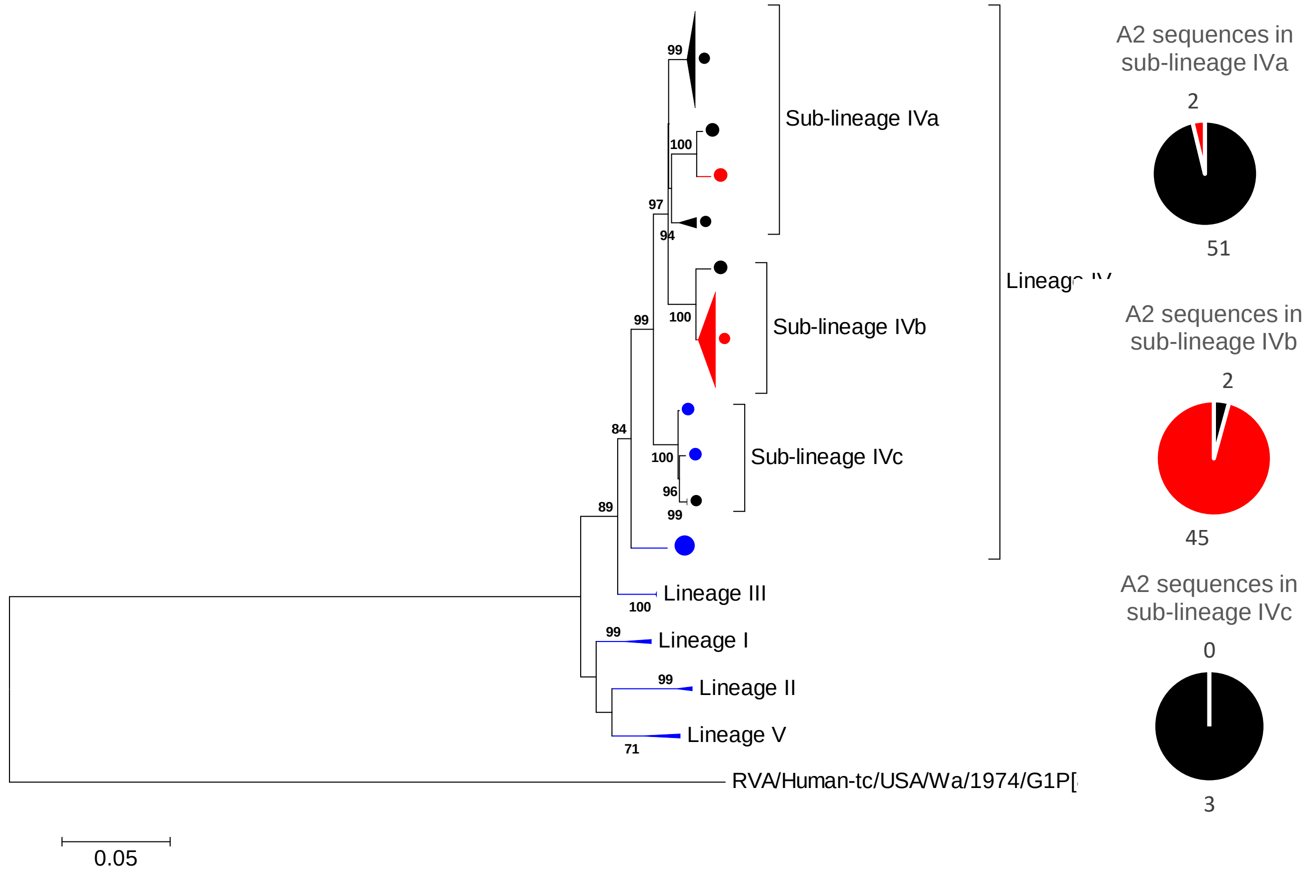

Figure S5: Maximum likelihood phylogenetic tree based on the ORF of the genome segment 5 encoding NSP1. The T92 + G evolutionary model was used for phylogenetic inference. South African pre-vaccine A2 sequences are highlighted in indicated with black circles while post-vaccine sequences are highlighted in red circles. The pie-chart summarizes the number of pre- and post-vaccine sequences. Lineages are indicated in roman numerals. Only bootstrap values ≥ 70% are shown adjacent to each branch node. Scale bar indicates the number of nucleotide substitutions per site.

NSP2

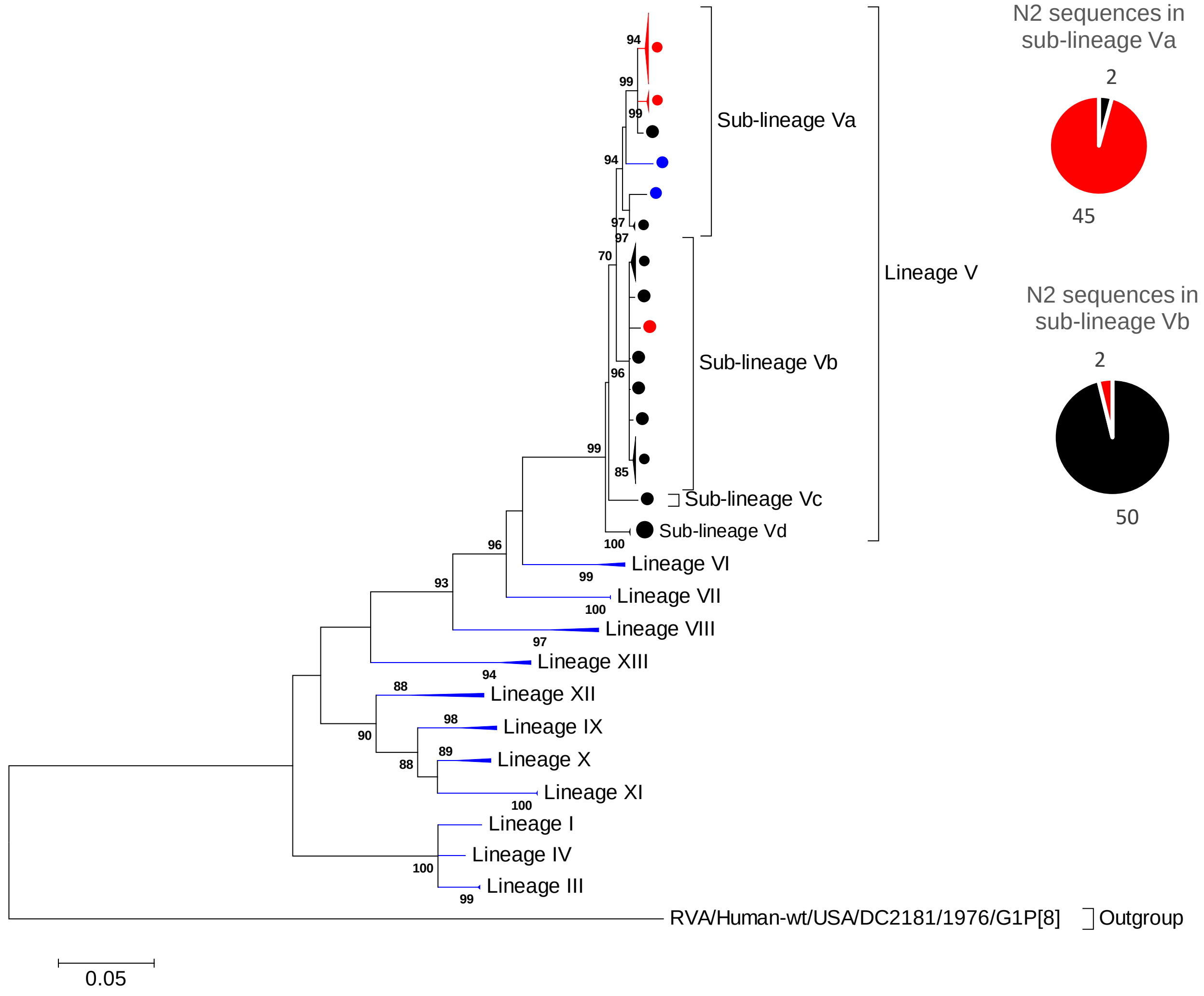

Figure S6: Maximum likelihood phylogenetic tree based on the ORF of the genome segment 8 encoding NSP2. The T92 + G evolutionary model was used for phylogenetic inference. South African pre-vaccine N2 sequences are indicated with black circles while post-vaccine sequences are highlighted in red circles. The reference sequences that were used to construct the tree are indicated with blue circle. The pie-chart summarizes the number of pre- and post-vaccine sequences. Lineages are indicated in roman numerals. Only bootstrap values ≥ 70% are shown adjacent to each branch node. Scale bar indicates the number of nucleotide substitutions per site.

NSP3

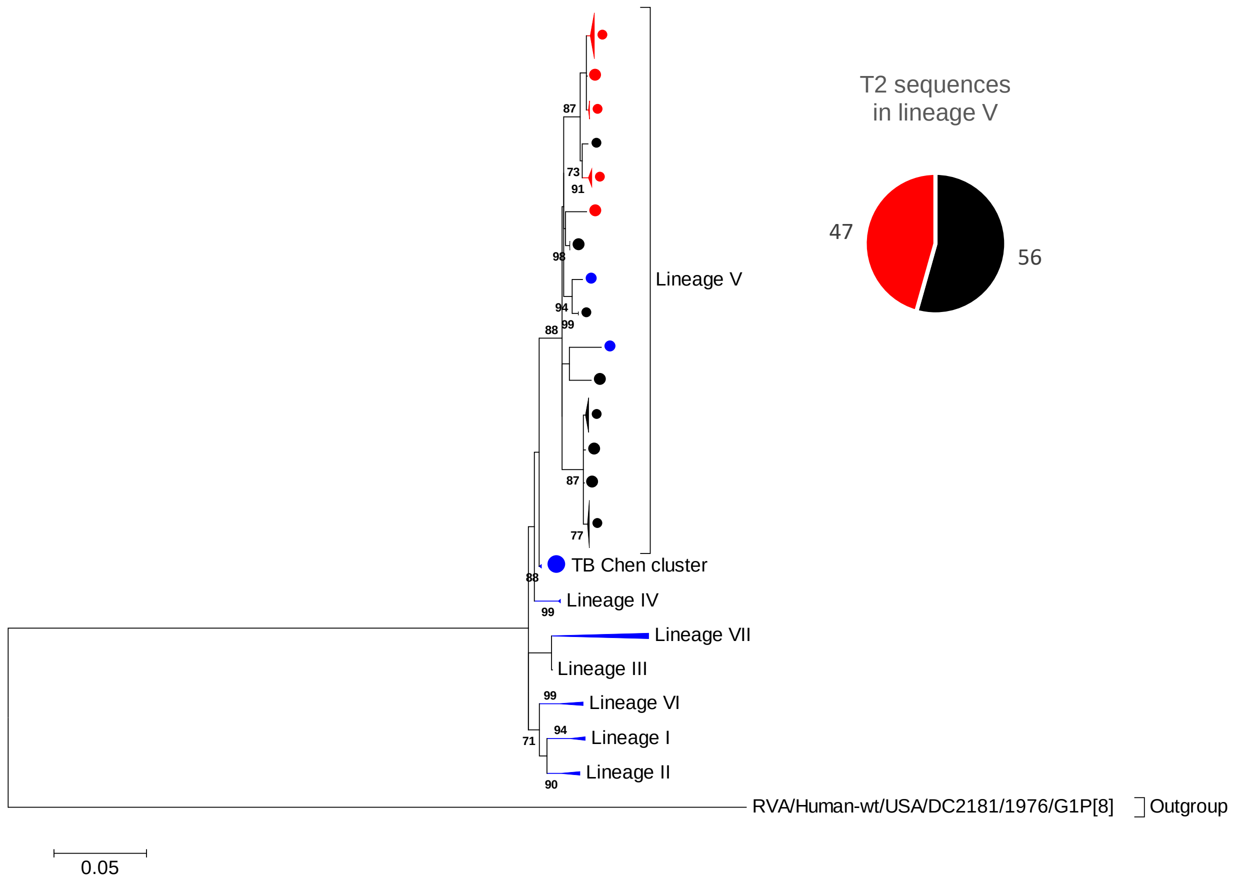

Figure S7: Maximum likelihood phylogenetic tree based on the ORF of the genome segment 7 encoding NSP3. The T92 + G evolutionary model was used for phylogenetic inference. South African pre-vaccine T2 sequences are indicated with black circles while post-vaccine sequences are highlighted in red circles. The reference sequences that were used to construct the tree are indicated with blue circles. The pie-chart summarizes the number of pre- and post-vaccine sequences. Lineages are indicated in roman numerals. Only bootstrap values ≥ 70% are shown adjacent to each branch node. Scale bar indicates the number of nucleotide substitutions per site.

NSP4

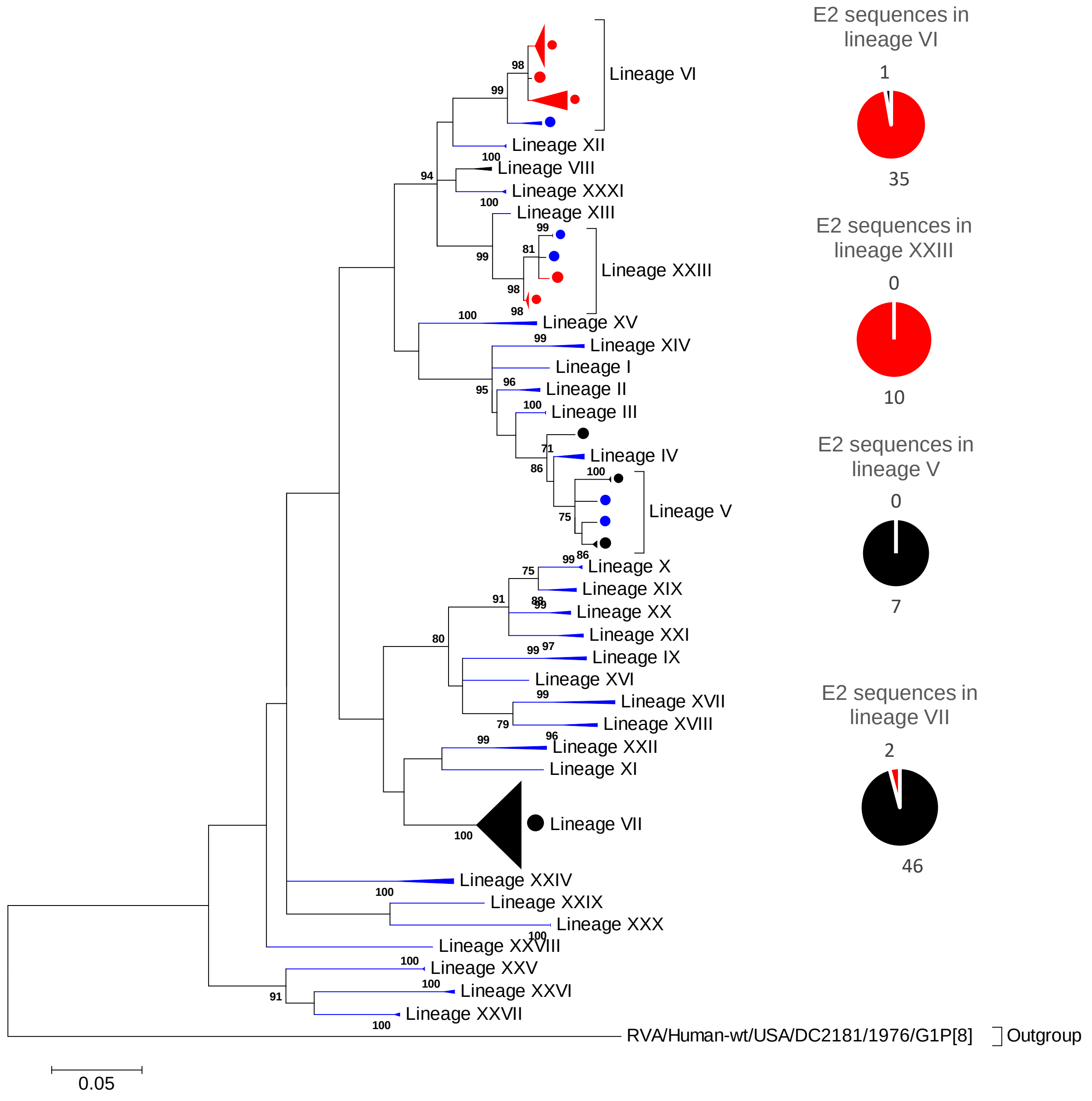

Figure S8: Maximum likelihood phylogenetic tree based on the ORF of the genome segment 10 encoding NSP4. The T92+G+I evolutionary model was used for phylogenetic inference. South African pre-vaccine E2 sequences are highlighted in black circles while post-vaccine sequences are highlighted in red circles. The reference sequences that were used to construct the tree are indicated with blue circles. The pie-chart summarizes the number of pre- and post-vaccine sequences. Lineages are indicated in roman numerals. Only bootstrap values ≥ 70% are shown adjacent to each branch node. Scale bar indicates the number of nucleotide substitutions per site.

NSP5

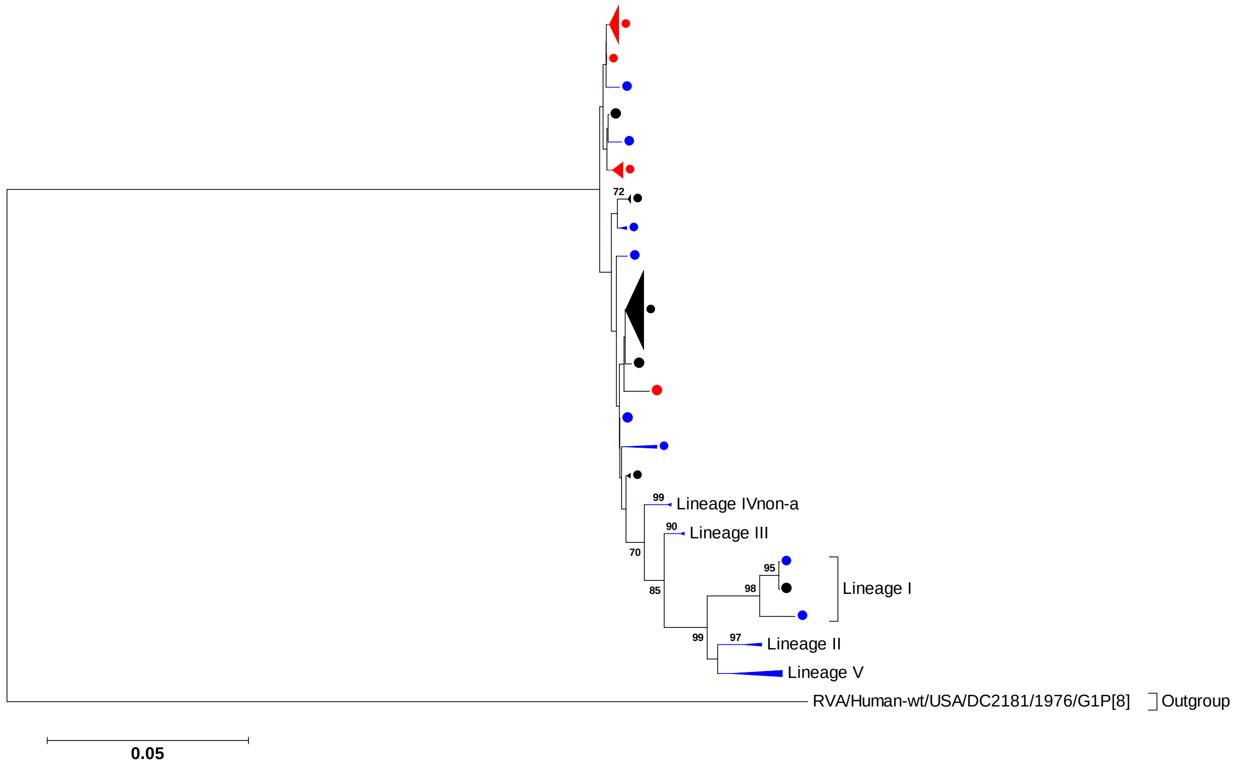

Figure S9: Maximum likelihood phylogenetic tree based on the ORF of the genome segment 11 encoding NSP5. The T92 + G evolutionary model was used for phylogenetic inference. South African pre-vaccine H2 sequences are highlighted in black circles while post-vaccine sequences are highlighted in red circles. The reference sequences that were used to construct the tree are indicated with blue circles. Lineages are indicated in roman numerals. Only bootstrap values ≥ 70% are shown adjacent to each branch node. Scale bar indicates the number of nucleotide substitutions per site.

Table S1: Distribution of South African G2P[4] stool specimens by year

	Pre-vaccine period						Post-vaccine period					
**Year**	**2003**	**2006**	**2007**	**2008**	**2009**	**2010**	**2012**	**2013**	**2014**	**2015**	**2016**	**2017**
**Numbers**	**6**	**1**	**9**	**13**	**13**	**10**	**11**	**15**	**12**	**4**	**3**	**1**

The rotavirus positive stool specimens were previously characterized as part of the WHO-coordinated RVA surveillance network. The G2P[4] specimens were unavailable in archival storage for the years 2002, 2004, 2005 and 2011. Pre-vaccine strains are from 2003-2010 while post-vaccine strains are from 2012-2017.

Table S2: Whole-genome constellations of 103 South African G2P[4] rotavirus A strains

	Gene segment	VP7	VP[4]	VP6	VP1	VP2	VP3	NSP1	NSP2	NSP3	NSP4	NSP5
	**Base pair size for full length sequences**	1062	2359	1356	3302	2684	2591	1563	1059	1064	751	821
	**Base pair size for open reading frame (ORF) sequences**	978	2325	1191	3264	2637	2505	1458	951	930	525	600
	**Average reads per gene segment**	22,982	54,187	27,226	75,439	55,035	59,540	26,912	15,693	18,338	15,561	11,994
	**Average coverage per gene segment**	5,077	7,528	5,359	6,889	6,065	5,321	4,137	4,392	4,443	5,020	3,509
							**Genotype constellations**					
	**Sequenced in this study**											
	Pre-vaccine strains											
1	**RVA/Human-wt/ZAF/UFS-NGS-MRC-DPRU2123/2003/G2P[4]**	G2	P[4]	I2	R2	C2	M2	A2	N2	T2	E2	H2
2	**RVA/Human-wt/ZAF/UFS-NGS-MRC-DPRU531/2003/G2P[4]**	G2	P[4]	I2	R2	C2	M2	A2	N2	T2	E2	H2
3	**RVA/Human-wt/ZAF/UFS-NGS-MRC-DPRU580/2003/G2P[4]**	G2	P[4]	I2	R2	C2	M2	A2	N2	T2	E2	H2
4	**RVA/Human-wt/ZAF/UFS-NGS-MRC-DPRU594/2003/G2P[4]**	G2	P[4]	I2	R2	C2	M2	A2	N2	T2	E2	H2
5	**RVA/Human-wt/ZAF/UFS-NGS-MRC-DPRU603/2003/G2P[4]**	G2	P[4]	I2	R2	C2	M2	A2	N2	T2	E2	H2
6	**RVA/Human-wt/ZAF/UFS-NGS-MRC-DPRU667/2003/G2P[4]**	G2	P[4]	I2	R2	C2	M2	A2	N2	T2	E2	H2
7	**RVA/Human-wt/ZAF/UFS-NGS-MRC-DPRU764/2006/G2P[4]**	G2	P[4]	I2	R2	C2	M2	A2	N2	T2	E2	H2
8	**RVA/Human-wt/ZAF/UFS-NGS-NICD150/2007G2P[4]**	G2	P[4]	I2	R2	C2	M2	A2	N2	T2	E2	H2
9	**RVA/Human-wt/ZAF/UFS-NGS-NICD419/2007/G2P[4]**	G2	P[4]	I2	R2	C2	M2	A2	N2	T2	E2	H2
10	**RVA/Human-wt/ZAF/UFS-NGS-NICD516/2007/G2P[4]**	G2	P[4]	I2	R2	C2	M2	A2	N2	T2	E2	H2
11	**RVA/Human-wt/ZAF/UFS-NGS-NICD582/2007/G2P[4]**	G2	P[4]	I2	R2	C2	M2	A2	N2	T2	E2	H2
12	**RVA/Human-wt/ZAF/UFS-NGS-NICD626/2007/G2P[4]**	G2	P[4]	I2	R2	C2	M2	A2	N2	T2	E2	H2
13	**RVA/Human-wt/ZAF/UFS-NGS-NICD673/2007/G2P[4]**	G2	P[4]	I2	R2	C2	M2	A2	N2	T2	E2	H2
14	**RVA/Human-wt/ZAF/UFS-NGS-NICD759/2007/G2P[4]**	G2	P[4]	I2	R2	C2	M2	A2	N2	T2	E2	H2
15	**RVA/Human-wt/ZAF/UFS-NGS-MRC-DPRU1271/2007/G2P[4]**	G2	P[4]	I2	R2	C2	M2	A2	N2	T2	E2	H2
16	**RVA/Human-wt/ZAF/UFS-NGS-MRC-DPRU1285/2007/G2P[4]**	G2	P[4]	I2	R2	C2	M2	A2	N2	T2	E2	H2
17	**RVA/Human-wt/ZAF/UFS-NGS-NICD1079/2008/G2P[4]**	G2	P[4]	I2	R2	C2	M2	A2	N2	T2	E2	H2
18	**RVA/Human-wt/ZAF/UFS-NGS-NICD1112/2008/G2P[4]**	G2	P[4]	I2	R2	C2	M2	A2	N2	T2	E2	H2

**19**	**RVA/Human-wt/ZAF/UFS-NGS-NICD1122/2008/G2P[4]**	G2	P[4]	I2	R2	C2	M2	A2	N2	T2	E2	H2
**20**	**RVA/Human-wt/ZAF/UFS-NGS-NICD1138/2008/G2P[4]**	G2	P[4]	I2	R2	C2	M2	A2	N2	T2	E2	H2
**21**	**RVA/Human-wt/ZAF/UFS-NGS-NICD1165/2008/G2P[4]**	G2	P[4]	I2	R2	C2	M2	A2	N2	T2	E2	H2
**22**	**RVA/Human-wt/ZAF/UFS-NGS-NICD1180/2008/G2P[4]**	G2	P[4]	I2	R2	C2	M2	A2	N2	T2	E2	H2
**23**	**RVA/Human-wt/ZAF/UFS-NGS-NICD1355/2008/G2P[4]**	G2	P[4]	I2	R2	C2	M2	A2	N2	T2	E2	H2
**24**	**RVA/Human-wt/ZAF/UFS-NGS-MRC-DPRU985/2008/G2P[4]**	G2	P[4]	I2	R2	C2	M2	A2	N2	T2	E2	H2
**25**	**RVA/Human-wt/ZAF/UFS-NGS-MRC-DPRU1036/2008/G2P[4]**	G2	P[4]	I2	R2	C2	M2	A2	N2	T2	E2	H2
**26**	**RVA/Human-wt/ZAF/UFS-NGS-MRC-DPRU1040/2008/G2P[4]**	G2	P[4]	I2	R2	C2	M2	A2	N2	T2	E2	H2
**27**	**RVA/Human-wt/ZAF/UFS-NGS-MRC-DPRU1041/2008/G2P[4]**	G2	P[4]	I2	R2	C2	M2	A2	N2	T2	E2	H2
**28**	**RVA/Human-wt/ZAF/UFS-NGS-MRC-DPRU1058/2008/G2P[4]**	G2	P[4]	I2	R2	C2	M2	A2	N2	T2	E2	H2
**29**	**RVA/Human-wt/ZAF/UFS-NGS-MRC-DPRU1901/2008/G2P[4]**	G2	P[4]	I2	R2	C2	M2	A2	N2	T2	E2	H2
**30**	**RVA/Human-wt/ZAF/UFS-NGS-NICD3532/2009/G2P[4]**	G2	P[4]	I2	R2	C2	M2	A2	N2	T2	E2	H2
**31**	**RVA/Human-wt/ZAF/UFS-NGS-NICD3354/2009/G2P[4]**	G2	P[4]	I2	R2	C2	M2	A2	N2	T2	E2	H2
**32**	**RVA/Human-wt/ZAF/UFS-NGS-NICD4207/2009/G2P[4]**	G2	P[4]	I2	R2	C2	M2	A2	N2	T2	E2	H2
**33**	**RVA/Human-wt/ZAF/UFS-NGS-NICD4012/2009/G2P[4]**	G2	P[4]	I2	R2	C2	M2	A2	N2	T2	E2	H2
**34**	**RVA/Human-wt/ZAF/UFS-NGS-NICD3681/2009/G2P[4]**	G2	P[4]	I2	R2	C2	M2	A2	N2	T2	E2	H2
**35**	**RVA/Human-wt/ZAF/UFS-NGS-NICD3518/2009/G2P[4]**	G2	P[4]	I2	R2	C2	M2	A2	N2	T2	E2	H2
**36**	**RVA/Human-wt/ZAF/UFS-NGS-NICD3446/2009/G2P[4]**	G2	P[4]	I2	R2	C2	M2	A2	N2	T2	E2	H2
**37**	**RVA/Human-wt/ZAF/UFS-NGS-NICD3442/2009/G2P[4]**	G2	P[4]	I2	R2	C2	M2	A2	N2	T2	E2	H2
**38**	**RVA/Human-wt/ZAF/UFS-NGS-MRC-DPRU1071/2009/G2P[4]**	G2	P[4]	I2	R2	C2	M2	A2	N2	T2	E2	H2
**39**	**RVA/Human-wt/ZAF/UFS-NGS-MRC-DPRU1077/2009/G2P[4]**	G2	P[4]	I2	R2	C2	M2	A2	N2	T2	E2	H2
**40**	**RVA/Human-wt/ZAF/UFS-NGS-MRC-DPRU1097/2009/G2P[4]**	G2	P[4]	I2	R2	C2	M2	A2	N2	T2	E2	H2
**41**	**RVA/Human-wt/ZAF/UFS-NGS-MRC-DPRU2288/2009/G2P[4]**	G2	P[4]	I2	R2	C2	M2	A2	N2	T2	E2	H2
**42**	**RVA/Human-wt/ZAF/UFS-NGS-MRC-DPRU2326/2009/G2P[4]**	G2	P[4]	I2	R2	C2	M2	A2	N2	T2	E2	H2
**43**	**RVA/Human-wt/ZAF/UFS-NGS-NICD6388/2010/G2P[4]**	G2	P[4]	I2	R2	C2	M2	A2	N2	T2	E2	H2
**44**	**RVA/Human-wt/ZAF/UFS-NGS-NICD6171/2010/G2P[4]**	G2	P[4]	I2	R2	C2	M2	A2	N2	T2	E2	H2
**45**	**RVA/Human-wt/ZAF/UFS-NGS-NICD6150/2010/G2P[4]**	G2	P[4]	I2	R2	C2	M2	A2	N2	T2	E2	H2
**46**	**RVA/Human-wt/ZAF/UFS-NGS-NICD6099/2010/G2P[4]**	G2	P[4]	I2	R2	C2	M2	A2	N2	T2	E2	H2
**47**	**RVA/Human-wt/ZAF/UFS-NGS-NICD5884/2010/G2P[4]**	G2	P[4]	I2	R2	C2	M2	A2	N2	T2	E2	H2
**48**	**RVA/Human-wt/ZAF/UFS-NGS-NICD5625/2010/G2P[4]**	G2	P[4]	I2	R2	C2	M2	A2	N2	T2	E2	H2
**49**	**RVA/Human-wt/ZAF/UFS-NGS-MRC-DPRU1473/2010/G2P[4]**	G2	P[4]	I2	R2	C2	M2	A2	N2	T2	E2	H2

**50**	**RVA/Human-wt/ZAF/UFS-NGS-MRC-DPRU1507/2010/G2P[4]**	G2	P[4]	I2	R2	C2	M2	A2	N2	T2	E2	H2
**51**	**RVA/Human-wt/ZAF/UFS-NGS-MRC-DPRU1510/2010/G2P[4]**	G2	P[4]	I2	R2	C2	M2	A2	N2	T2	E2	H2
**52**	**RVA/Human-wt/ZAF/UFS-NGS-MRC-DPRU1520/2010/G2P[4]**	G2	P[4]	I2	R2	C2	M2	A2	N2	T2	E2	H2
	Post-vaccine strains											
**53**	**RVA/Human-wt/ZAF/UFS-NGS-NICD8873/2012/G2P[4]**	G2	P[4]	I2	R2	C2	M2	A2	N2	T2	E2	H2
**54**	**RVA/Human-wt/ZAF/UFS-NGS-NICD9659/2012/G2P[4]**	G2	P[4]	I2	R2	C2	M2	A2	N2	T2	E2	H2
**55**	**RVA/Human-wt/ZAF/UFS-NGS-NICD9554/2012/G2P[4]**	G2	P[4]	I2	R2	C2	M2	A2	N2	T2	E2	H2
**56**	**RVA/Human-wt/ZAF/UFS-NGS-NICD9335/2012/G2P[4]**	G2	P[4]	I2	R2	C2	M2	A2	N2	T2	E2	H2
**57**	**RVA/Human-wt/ZAF/UFS-NGS-NICD9330/2012/G2P[4]**	G2	P[4]	I2	R2	C2	M2	A2	N2	T2	E2	H2
**58**	**RVA/Human-wt/ZAF/UFS-NGS-NICD9329/2012/G2P[4]**	G2	P[4]	I2	R2	C2	M2	A2	N2	T2	E2	H2
**59**	**RVA/Human-wt/ZAF/UFS-NGS-NICD9171/2012/G2P[4]**	G2	P[4]	I2	R2	C2	M2	A2	N2	T2	E2	H2
**60**	**RVA/Human-wt/ZAF/UFS-NGS-NICD9045/2012/G2P[4]**	G2	P[4]	I2	R2	C2	M2	A2	N2	T2	E2	H2
**61**	**RVA/Human-wt/ZAF/UFS-NGS-NICD9040/2012/G2P[4]**	G2	P[4]	I2	R2	C2	M2	A2	N2	T2	E2	H2
**62**	**RVA/Human-wt/ZAF/UFS-NGS-NICD9031/2012/G2P[4]**	G2	P[4]	I2	R2	C2	M2	A2	N2	T2	E2	H2
**63**	**RVA/Human-wt/ZAF/UFS-NGS-NICD8940/2012/G2P[4]**	G2	P[4]	I2	R2	C2	M2	A2	N2	T2	E2	H2
**64**	**RVA/Human-wt/ZAF/UFS-NGS-NICD12041/2013/G2P[4]**	G2	P[4]	I2	R2	C2	M2	A2	N2	T2	E2	H2
**65**	**RVA/Human-wt/ZAF/UFS-NGS-NICD10248/2013/G2P[4]**	G2	P[4]	I2	R2	C2	M2	A2	N2	T2	E2	H2
**66**	**RVA/Human-wt/ZAF/UFS-NGS-MRC-DPRU60/2013/G2P[4]**	G2	P[4]	I2	R2	C2	M2	A2	N2	T2	E2	H2
**67**	**RVA/Human-wt/ZAF/UFS-NGS-MRC-DPRU68/2013/G2P[4]**	G2	P[4]	I2	R2	C2	M2	A2	N2	T2	E2	H2
**68**	**RVA/Human-wt/ZAF/UFS-NGS-MRC-DPRU75/2013/G2P[4]**	G2	P[4]	I2	R2	C2	M2	A2	N2	T2	E2	H2
**69**	**RVA/Human-wt/ZAF/UFS-NGS-MRC-DPRU81/2013/G2P[4]**	G2	P[4]	I2	R2	C2	M2	A2	N2	T2	E2	H2
**70**	**RVA/Human-wt/ZAF/UFS-NGS-MRC-DPRU84/2013/G2P[4]**	G2	P[4]	I2	R2	C2	M2	A2	N2	T2	E2	H2
**71**	**RVA/Human-wt/ZAF/UFS-NGS-MRC-DPRU182/2013/G2P[4]**	G2	P[4]	I2	R2	C2	M2	A2	N2	T2	E2	H2
**72**	**RVA/Human-wt/ZAF/UFS-NGS-MRC-DPRU185/2013/G2P[4]**	G2	P[4]	I2	R2	C2	M2	A2	N2	T2	E2	H2
**73**	**RVA/Human-wt/ZAF/UFS-NGS-MRC-DPRU198/2013/G2P[4]**	G2	P[4]	I2	R2	C2	M2	A2	N2	T2	E2	H2
**74**	**RVA/Human-wt/ZAF/UFS-NGS-MRC-DPRU200/2013/G2P[4]**	G2	P[4]	I2	R2	C2	M2	A2	N2	T2	E2	H2
**75**	**RVA/Human-wt/ZAF/UFS-NGS-MRC-DPRU203/2013/G2P[4]**	G2	P[4]	I2	R2	C2	M2	A2	N2	T2	E2	H2
**76**	**RVA/Human-wt/ZAF/UFS-NGS-MRC-DPRU975/2013/G2P[4]**	G2	P[4]	I2	R2	C2	M2	A2	N2	T2	E2	H2
**77**	**RVA/Human-wt/ZAF/UFS-NGS-MRC-DPRU978/2013/G2P[4]**	G2	P[4]	I2	R2	C2	M2	A2	N2	T2	E2	H2
**78**	**RVA/Human-wt/ZAF/UFS-NGS-MRC-DPRU986/2013/G2P[4]**	G2	P[4]	I2	R2	C2	M2	A2	N2	T2	E2	H2
**79**	**RVA/Human-wt/ZAF/UFS-NGS-NICD13907/2014/G2P[4]**	G2	P[4]	I2	R2	C2	M2	A2	N2	T2	E2	H2

**80**	**RVA/Human-wt/ZAF/UFS-NGS-NICD13878/2014/G2P[4]**	G2	P[4]	I2	R2	C2	M2	A2	N2	T2	E2	H2
**81**	**RVA/Human-wt/ZAF/UFS-NGS-NICD13791/2014/G2P[4]**	G2	P[4]	I2	R2	C2	M2	A2	N2	T2	E2	H2
**82**	**RVA/Human-wt/ZAF/UFS-NGS-NICD13522/2014/G2P[4]**	G2	P[4]	I2	R2	C2	M2	A2	N2	T2	E2	H2
**83**	**RVA/Human-wt/ZAF/UFS-NGS-NICD13335/2014/G2P[4]**	G2	P[4]	I2	R2	C2	M2	A2	N2	T2	E2	H2
**84**	**RVA/Human-wt/ZAF/UFS-NGS-NICD13333/2014/G2P[4]**	G2	P[4]	I2	R2	C2	M2	A2	N2	T2	E2	H2
**85**	**RVA/Human-wt/ZAF/UFS-NGS-NICD13083/2014/G2P[4]**	G2	P[4]	I2	R2	C2	M2	A2	N2	T2	E2	H2
**86**	**RVA/Human-wt/ZAF/UFS-NGS-NICD13081/2014/G2P[4]**	G2	P[4]	I2	R2	C2	M2	A2	N2	T2	E2	H2
**87**	**RVA/Human-wt/ZAF/UFS-NGS-NICD12838/2014/G2P[4]**	G2	P[4]	I2	R2	C2	M2	A2	N2	T2	E2	H2
**88**	**RVA/Human-wt/ZAF/UFS-NGS-NICD12832/2014/G2P[4]**	G2	P[4]	I2	R2	C2	M2	A2	N2	T2	E2	H2
**89**	**RVA/Human-wt/ZAF/UFS-NGS-NICD12795/2014/G2P[4]**	G2	P[4]	I2	R2	C2	M2	A2	N2	T2	E2	H2
**90**	**RVA/Human-wt/ZAF/UFS-NGS-MRC-DPRU952/2014/G2P[4]**	G2	P[4]	I2	R2	C2	M2	A2	N2	T2	E2	H2
**91**	**RVA/Human-wt/ZAF/UFS-NGS-NICD15385/2015/G2P[4]**	G2	P[4]	I2	R2	C2	M2	A2	N2	T2	E2	H2
**92**	**RVA/Human-wt/ZAF/UFS-NGS-NICD15070/2015/G2P[4]**	G2	P[4]	I2	R2	C2	M2	A2	N2	T2	E2	H2
**93**	**RVA/Human-wt/ZAF/UFS-NGS-NICD15034/2015/G2P[4]**	G2	P[4]	I2	R2	C2	M2	A2	N2	T2	E2	H2
**94**	**RVA/Human-wt/ZAF/UFS-NGS-NICD14647/2015/G2P[4]**	G2	P[4]	I2	R2	C2	M2	A2	N2	T2	E2	H2
**95**	**RVA/Human-wt/ZAF/UFS-NGS-NICD17155/2016/G2P[4]**	G2	P[4]	I2	R2	C2	M2	A2	N2	T2	E2	H2
**96**	**RVA/Human-wt/ZAF/UFS-NGS-NICD16983/2016/G2P[4]**	G2	P[4]	I2	R2	C2	M2	A2	N2	T2	E2	H2
**97**	**RVA/Human-wt/ZAF/UFS-NGS-MRC-DPRU12323/2016/G2P[4]**	G2	P[4]	I2	R2	C2	M2	A2	N2	T2	E2	H2
**98**	**RVA/Human-wt/ZAF/UFS-NGS-NICD18920/2017/G2P[4]**	G2	P[4]	I2	R2	C2	M2	A2	N2	T2	E2	H2
	**Acquired from GenBank**											
	Pre-vaccine strains											
99	**RVA/Human-wt/ZAF/MRC-DPRU618/2003/G2P[4]**	G2	P[4]	I2	R2	C2	M2	A2	N2	T2	E2	H2
**100**	**RVA/Human-wt/ZAF/MRC-DPRU81/2007/G2P[4]**	G2	P[4]	I2	R2	C2	M2	A2	N2	T2	E2	H2
101	**RVA/Human-wt/ZAF/MRC-DPRU1061/2009/G2P[4]**	G2	P[4]	I2	R2	C2	M2	A2	N2	T2	E2	H2
**102**	**RVA/Human-wt/ZAF/3203WC/2009/G2P[4]**	G2	P[4]	I2	R2	C2	M2	A2	N2	T2	E2	H2
	Post-vaccine strain											
**103**	**RVA/Human-wt/ZAF/MRC-DPRU82/2012/G2P[4]**	G2	P[4]	I2	R2	C2	M2	A2	N2	T2	E2	H2

Table S3: Nucleotide identity analysis between South African pre- and post-vaccine G2P[4] strains

Gene segment	VP7 (S9)	VP4 (S4)	VP6 (S6)	VP1 (S1)	VP2 (S2)	VP3 (S3)	NSP1 (S5)	NSP2 (S8)	NSP3 (S7)	NSP4 (S10)	NSP5 (S11)
**Comparison between pre- and post-vaccine G2P[4] strains**	95.1-100	92.0-100	96.3-100	91.0-100	96.6-100	87.1-100	95.7-100	95.6-100	95.7-100	85.3-100	94.3-100

A comparison between South African pre-vaccine G2P[4] strains with post-vaccine G2P[4] strains. The NT identity values are in percentage. The segment number is indicated in brackets adjacent to each viral protein.

Tables S4-S9

Table S4: Amino acid differences and frequency between pre- and post-vaccination VP1 sequences

**Gene segment**		**VP1**				
**Amino acid site**		**56**	**159**	**293**	**294**	**432**
**Region**		**N-terminal domain**				**Central polymerase domain**
**Pre-vaccine period (2003-2010)**	**Amino acid residue and frequency of occurrence**	K (55/56)	K (51/56)	N (51/56)	S(50/56)	R (54/56)
**Post-vaccine period (2012-2017)**		R (33/47)	R (45/47)	D (45/47)	N(45/47)	K(35/47)

The N-terminal domain of RVA VP1 facilitates entry of ssRNA templates into the catalytic center and also in sequence-specific recognition of viral RNAs (Lu et al. 2008; Ogden et al. 2011). The central polymerase domain is involved in replication and transcription (Lu et al. 2008).

Lu X, McDonald SM, Tortorici MA, Tao YJ, Vasquez-Del Carpio R, Nibert ML, Patton JT, Harrison SC. Mechanism for coordinated RNA packaging and genome replication by rotavirus polymerase VP1. *Structure* 2008; 16:1678-88. doi: 10.1016/j.str.2008.09.006.

Ogden KM, Ramanathan HN, Patton JT. Residues of the rotavirus RNA-dependent RNA polymerase template entry tunnel that mediate RNA recognition and genome replication. *J Virol* 2011; 85:1958-69. doi: 10.1128/JVI.01689-10.

Table S5: Amino acid differences and frequency between pre- and post-vaccination VP3 sequences

Gene segment		VP3													
**Amino acid site**		**28**	**49**	**50**	**69**	**88**	**107**	**109**	**132**	**143**	**161**	**167**	**204**	**209**	**222**
**Region**		N-terminal domain											Guanine-N7-Methyltransferase (N7-MTase) domain		
**Pre-vaccine period (2003-2010)**	**Amino acid residue and frequency of occurrence**	E (54/56)	N (54/56)	V (54/56)	I (54/56)	V (54/56)	H (54/56)	I (54/56)	S (54/56)	R (54/56)	D (54/56)		D (53/56)	I (54/56)	Y (54/56)
**Post-vaccine period (2012-2017)**		D (35/47)	S (34/47)	I (34/47)	M (45/47)	I (35/47)	Y (35/47)	V (35/47)	N (35/47)	K (35/47)	N (45/47)	N (35/47)	S (35/47)	V (34/47)	H (35/47)

Gene segment		VP3													
**Amino acid site**		**226**	**274**	**275**	**277**	**281**	**282**	**316**	**326**	**327**	**347**	**348**	**355**	**356**	**359**
**Region**		N7-MTase domain	2'-*O*-Methyltransfrase (2'-*O*-MTase) domain												
**Pre-vaccine period (2003-2010)**	**Amino acid residue and frequency of occurrence**	E (54/56)	N (54/56)	V (54/56)	I (54/56)	V (54/56)	V (54/56)	N (54/56)	N (55/56)	D (54/56)	T (54/56)	V (52/56)	K (54/56)	I (50/56)	R (54/46)
**Post-vaccine period (2012-2017)**		D (35/47)	S (34/47)	I (34/47)	M (45/47)	I (35/47)	I (35/47)	D (35/47)	D (35/47)	N (35/47)	N (35/47)<br>P (10/47)	M (35/47)	R (35/47)	V (32/47)	E (35/47)

Gene segment

		VP3													
	**Amino acid site**	363	379	412	425	437	458	459	468	518	605	687	688	703	730
	**Region**	2’-*O*-Methyltransferase (2’-*O*-MTase) domain				Guanine-N7-methyltransferase (N7-MTase) domain					Guanylyltransferase (GTase)/ RNA 5’-triphosphatase (RTPase) domain			2’-5’-Phosphodiesterase (PDE) domain	
**Pre-vaccine period (2003-2010)**	**Amino acid residue and frequency of occurrence**	D (54/56)	V (54/56)	V (53/56)	I (54/56)	T (54/56)	I (54/46)	I (54/56)	I (50/56)	N (53/56)	V (55/56)	K (54/56)	V (55/56)	V (53/56)	M (47/56)
**Post-vaccine period (2012-2017)**		S (35/47)	I (35/47)	I (35/47)	V (34/47)	I (35/47)	V (35/47)	T (35/47)	F (45/47)	S (35/47)	I (35/47)	R (35/47)	A (35/47)	(36/47)	I (35/47)

Gene segment		VP3						
**Amino acid site**		**734**	**736**	**740**	**749**	**767**	**798**	**816**
**Region**		2’5’-Phosphodiesterase (PDE) domain						
**Pre-vaccine period (2003-2010)**	**Amino acid residue and frequency of occurrence**	V (54/56)	T(54/56)	R (53/56)	E (54/56)	I (54/56)	V (53/56)	Y (53/56)
**Post-vaccine period (2012-2017)**		A(35/47)	A(31/47)	K(32/47)	D(35/47)	V(35/47)	I (35/47)	H (34/47)

The guanine-N7-methyltransferase (N7-Mtase) domain is involved in methylation of the guanine cap, 2’-O-methyltransferase (2’-O-MTase) methylates the ribose of the initiating nucleotide, guanylyltransferase (GTase) conjugates GMP to the mRNA through 5’-5’ linkage, RNA 5’-triphosphatase (RTPase) reomves the ʏ-phosphate from the 5’ end of the mRNA while 2’-5’-phosphodiesterase (PDE) domain is involved in antagonization of antiviral innate immunity through inhibition of the oligoadenylate synthase/ RNase pathyway (Zhang et al. 2013; Ogden et al. 2014).

Ogden KM, Snyder MJ, Dennis AF, Patton JT. Predicted structure and domain organization of rotavirus capping enzyme and innate immune antagonist VP3. *J Virol* 2014: 15; 88:9072-9085. doi: 10.1128/JVI.00923-14.

Zhang R, Jha BK, Ogden KM, Dong B, Zhao L, Elliott R, Patton JT, Silverman RH, Weiss SR. Homologous 2′, 5′-phosphodiesterases from disparate RNA viruses antagonize antiviral innate immunity. *PNAS* 2013; 110:13114-13119.

Table S6: Amino acid differences and frequency between pre- and post-vaccination VP6 sequences

**Gene segment**		**VP6**
**Amino acid site**		**281**
**Region**		**Trimerization domain; hsc70 binding region**
**Pre-vaccine period (2003-2010)**	**Amino acid residue and frequency of occurrence**	V (55/56)
**Post-vaccine period (2012-2017)**		I (35/47)

The trimerization domain is required for VP6 assembly. Site 281 also falls within the region suggested to interact with hsc70(heat shock cognate protein). The hsc70 is a co-receptor for rotavirus entry into susceptible cells (Gualtero et al. 2007).

Gualtero DF, Guzman F, Acosta O, Guerrero CA. Amino acid domains 280–297 of VP6 and 531–554 of VP4 are implicated in heat shock cognate protein hsc70-mediated rotavirus infection. *Arch Virol* 2007; 152:2183-2196. doi: 10.1007/s00705-007-1055-5.

Table S7: Amino acid differences and frequency between pre- and post-vaccination NSP1 sequences

Gene segment		NSP1								
Amino acid site		103	234	246	273	319	333	334	429	464
Region		Cytoskeleton-localization domain				IRF-binding domain				
Pre-vaccine period (2003-2010)	Amino acid residue and frequency of occurrence	G (44/56)	N (54/56)	I (55/56)	L (54/56)	H (53/56)	W (54/56)	T (55/56)	K (55/56)	N (55/56)
Post-vaccine period (2012-2017)		D (44/57)	I (44/47)	L (25/47)	P (45/47)	R (44/47)	L (45/47)	N(25/47)	R (21/47)	T (25/47)

The NSP1 cytoskeleton binding domain enables the NSP1 to localize to the cytoplasm during infection and associate with the cytoskeleton (Hua et al. 1994). The interferon regulatory factor (IRF) binding domain is involved in inhibiting expression of type I interferon (Barro and Patton 2007).

Hua J, Chen X, Patton JT. Deletion mapping of the rotavirus metalloprotein NS53 (NSP1): the conserved cysteine-rich region is essential for virus-specific RNA binding. *J Virol* 1994;68:3990-4000. doi: 10.1128/JVI.68.6.3990-4000.1994.

Barro M, Patton JT. Rotavirus NSP1 inhibits expression of type I interferon by antagonizing the function of interferon regulatory factors IRF3, IRF5, and IRF7. *J Virol* 2007; 81:4473-4481.

Table S8: Amino acid differences and frequency between pre- and post-vaccination NSP2 sequences

Gene segment		NSP2			
**Amino acid site**		**42**	**74**	**75**	**82**
**Region**		**N-terminal domain**			**C-terminal domain**
**Pre-vaccine period (2003-2010)**	**Amino acid residue and frequency of occurrence**	K(55/56)	T (54/56)	V (47/56)	V (54/56)
**Post-vaccine period (2012-2017)**		R (21/47)	N (45/47)	A (45/47)	M (45/47)

The N and C-terminal domains are suggested to be involved in the sequence-independent RNA binding of NSP2 (Hu et al. 2012).

Hu L, Chow DC, Patton JT, Palzkill T, Estes MK, Prasad BV. Crystallographic analysis of rotavirus NSP2-RNA complex reveals specific recognition of 5′ GG sequence for RTPase activity. *J Virol* 2012; 86:10547-10557.

Table S9: Amino acid differences and frequency between pre- and post-vaccination NSP4 sequences

**Gene segment**		**NSP4**					
**Amino acid site**		**74**	**135**	**137**	**139**	**140**	**163**
**Region**		**Extracellular matrix protein-binding domain**	**Ca^{2+} binding and enterotoxin domain**	**Integrin binding domain**			**Tubulin binding domain**
**Pre-vaccine period (2003-2010)**	**Amino acid residue and frequency of occurrence**	T (55/56)	V (47/56)	R (52/56)	T (55/56)	N (52/56)	K (55/56)
**Post-vaccine period (2012-2017)**		A(34/47)	M (45/47)	Q (45/47)	I (35/47)	C (34/47)	R (45/47)

The extracelluar domain is involved interaction with extracelluar matrix during different infection stages (Boshuizen et al. 2014). The enterotoxin domain is implicated in diarrhea-induction through integrin binding and signaling (Seo et al. 2008). The integrin binding domain is involved in diarhroea induction through interaction with cellular plasma membrane integrin I domains (Seo et al. 2008).The tubulin binding domain is involved in binding of microtubules (Xu et al.2000) and acts as an intracellular receptor for double layered particles to facilitate infectious particle assembly (Au et al. 1989).

Boshuizen JA, Rossen JWA, Sitaram CK, Kimenai FFP, Simons-Oosterhuis Y, Laffeber C, Büller HA, Einerhand AWC. 2004. Rotavirus enterotoxin NSP4 binds to the extracellular matrix proteins laminin-β3 and fibronectin. J Virol. 78(18): 10045-10053.

Seo NS, Zeng CQY, Hyser JM, Utama B, Crawford SE, Kim KJ, Höök M, Estes MK. 2008. Integrins α1β1 and α2β1 are receptors for the rotavirus enterotoxin. PNAS.105(26): 8811-8818.

Xu A, Bellamy AR, Taylor JA. 2000. Immobilization of the early secretory pathway by a virus glycoprotein that binds to microtubules. EMBO J. 19(23): 6465-6474.

Au KS, Chan WK, Burns JW, Estes MK.1989. Receptor activity of rotavirus nonstructural glycoprotein NS28. J. Virol. *63*(11): 4553-4562
